# Precise Synthesis and Thermoresponsive Property of Poly(ethyl glycidyl ether) and Its Block and Statistic Copolymers with Poly(glycidol)

**DOI:** 10.3390/polym13223873

**Published:** 2021-11-09

**Authors:** Tingyu He, Yanqiu Wang, Atsushi Narumi, Liang Xu, Shin-ichiro Sato, Xiande Shen, Toyoji Kakuchi

**Affiliations:** 1 Research Center for Polymer Materials, School of Materials Science and Engineering, Changchun University of Science and Technology, Jilin 130022, China; 13019166435@163.com (T.H.); yqwang@cust.edu.cn (Y.W.); m13069984921@163.com (L.X.); 2 Graduate School of Organic Materials Science, Yamagata University, Yamagata 992-8510, Japan; narumi@yz.yamagata-u.ac.jp; 3 Division of Applied Chemistry, Faculty of Engineering, Hokkaido University, Hokkaido 060-8628, Japan; s-sato@eng.hokudai.ac.jp; 4 Chongqing Research Institute, Changchun University of Science and Technology, Chongqing 401135, China

**Keywords:** statistic copolymers, block copolymers, thermoresponsive polymers, LCST

## Abstract

In this paper, we describe a comprehensive study of the thermoresponsive properties of statistic copolymers and multiblock copolymers synthesized by poly(glycidol)s (PG) and poly(ethyl glycidyl ether) (PEGE) with different copolymerization methods. These copolymers were first synthesized by ring-opening polymerization (ROP), which was initiated by *tert*-butylbenzyl alcohol (*t*BBA) and 1-*tert*-butyl-4,4,4-tris(dimethylamino)-2,2-bis[tris(dimethylamino)phosphoranylidenamino]-2Λ^5^,4Λ^5^-catenadi(phosphazene) (*t*-Bu-P_4_) as the catalyst, and then the inherent protective groups were removed to obtain the copolymers without any specific chain end groups. The thermoresponsive property of the statistic copolymer PG*_x_-stat-*PEGE*_y_* was compared with the diblock copolymer PG*_x_*-*b*-PEGE*_y_*, and the triblock copolymers were compared with the pentablock copolymers. Among them, PG*-stat-*PEGE, PG-*b*-PEGE-*b*-PG*-b-*PEGE-*b*-PG, and PEGE-*b*-PG-*b*-PEGE-*b*-PG-*b*-PEGE, and even the specific ratio of PEGE-*b*-PG-*b*-PEGE, exhibited LCST-type phase transitions in water, which were characterized by cloud point (*T*_cp_). Although the ratio of x to y affected the value of the *T*_cp_ of PG_x_-*stat*-PEGE_y_, we found that the disorder of the copolymer has a decisive effect on the phase-transition behavior. The phase-transition behaviors of PG-*b*-PEGE, part of PEGE-*b*-PG-*b*-PEGE, and PG-*b*-PEGE-*b*-PG copolymers in water present a two-stage phase transition, that is, firstly LCST-type and then the upper critical solution temperature (UCST)-like phase transition. In addition, we have extended the research on the thermoresponsive properties of EGE homopolymers without specific α-chain ends.

## 1. Introduction

Aqueous solutions of some polymers show reversible phase-transition behavior, such as white turbidity when heated above the lower critical solution temperature (LCST) and dissolving again to clear when cooled below the LCST [1,2,3]. Such polymers include poly (*N*-substituted acrylamide)), poly (*N*-substituted methacrylamide), polyethers, and methyl cellulose [4,5,6,7,8]. On the other hand, sulfobetaine polymers, which are bipolar polymers, are known as polymers showing the upper critical solution temperature (UCST) after phase separation at low temperatures [9,10,11]. These polymers have been actively studied not only from the purely basic interest in clarifying phase-transition phenomena of aqueous solutions of polymers but also from the application aspect as intelligent polymers. The phase separation of poly (*N*-isopropylacrylamide) (PNIPAM) has been analyzed from various aspects using methods such as turbidimetry, calorimetry, nuclear magnetic resonance, fluorescence, light scattering, and neutron scattering [12,13,14]. It has been clarified by these studies that the macroscopic phase-separation phenomenon of PNIPAM is accompanied by the shrinkage phenomenon of polymer chains, called coil-globule transition [15,16]. That is to say, under LCST, polymer chains are dissolved by strong interactions between amide groups and water and take a statistic coil state [17]. However, when the hydrogen bond between amide groups and water is unstable due to the rise in water temperature and dehydration of the hydrophobic part occurs, polymer chains shrink to a globule state. In addition, it is important to clarify how the hydration state of each part of a polymer is changed by hydrophobic interaction. Since the infrared spectrum is sensitive to intermolecular interaction, a series of research was conducted by analyzing the hydration state of polymers using this method [15,18].

The clear-turbid phase transition occurred before and after cloud point temperature (*T*_cp_) [19,20,21,22,23]. The polymer hydration is thus wrapped by water molecules and dissolves when the temperature is lower than *T*_cp_. The dehydration reaction is accompanied by the entanglement of polymer chains when the temperature is higher than *T*_cp_, and its hydrophilicity, hydrophobicity, volume, micelle structure, etc., will change significantly as the temperature changes [24,25,26]. In recent years, poly(ethylene glycol) (PEG) [27], poly(*N*-isopropylacrylamide) (PNIPAM) [15], poly(poly-2-dimethylaminoethyl methacrylate) (PDMAEMA) [28], poly(*N*-vinyl caprolactam) (PNVCL) [29], poly(diethyl vinylphosphonate) (PDEVP) [30], and poly(oligoethylene glycol methacrylate) (POEGMA) [31], etc., have been widely studied.

To make the thermoresponsive polymer have a better application value, it is usually required that the polymer be equipped with an adjustable transition temperature in a wider temperature range. As has been established, linear thermoresponsive glycerol-polyethers can be excellent candidates due to their good biocompatibility and adjustability [32,33]. Poly(glycidyl ether)s, based on glycidyl methyl ether (GME) [34], ethoxyethyl glycidyl ether (EEGE) [35], and ethyl glycidyl ether (EGE) [35,36] exhibit thermoresponsive properties and undergo a coil-to-globule phase transition in water. Aoki et al. [35] detected that the phase transition temperature of these homopolymers is located at 14.6 °C for PEGE, 40.0 °C for PEEGE, and 57.7 °C for PGME and thus outside the physiologically relevant temperature range. To expand the scope of application of the polyglycerols, these water-soluble polyglycerol derivatives have received much attention due to their inherent biocompatible properties. Ogura et al. [37] synthesized the new amphiphilic diblock copolymers, which were named poly(ethyl glycidyl ether)-*block*-poly(ethylene oxide) (PEGE-*b*-PEO), and further aggregated the core shell into micelles at 40 °C, which is probably due to triggering by dehydration of the PEO chain segment. Interestingly, P(GME-*co*-EGE) copolymers with different GME/EGE ratios that have a cloud-point range from 10 °C to 60 °C prepared by Reinicke et al. [38] show similar behavior to those in the works of Weinhart et al. [39]. The poly(GME)-*stat*.-Co-(EGE) has an LCST at approximately 30 °C, with a monomer feed ratio of 1:3 (GME/EGE).

Hence, statistic copolymerization and block copolymerization are the general approaches used to adjust the ratio of two monomers to control the solubility of the polymer and select one or two temperature-sensitive monomers in order to design the *T*_cp_ of polymeric aggregates [40,41]. Previously, we expanded our precision BnGE-polymerization system to the synthesis of the “pristine” PG, as a glycerol-backbone polymeric intermediate, which can be post-modified to acquire a new thermoresponsive property of polymer PG*_n_*-EO*_m_*R’ [42]. Starting from here, we decided to design PG into the new thermoresponsive-property material by the incorporation of the more hydrophobic EGE comonomer using block and statistic copolymerization methods. In recent years, the development of structural versatility and the use of PG derivatives in biomedical applications have advanced by leaps and bounds [43]. The PG structure is very unique, with one hydroxyl group per repeated unit applicable, and the LCST can be induced by hydrophobic modification of the hydroxyl groups and provide a blueprint to utilize them in biomedical applications.

**Scheme 1:**
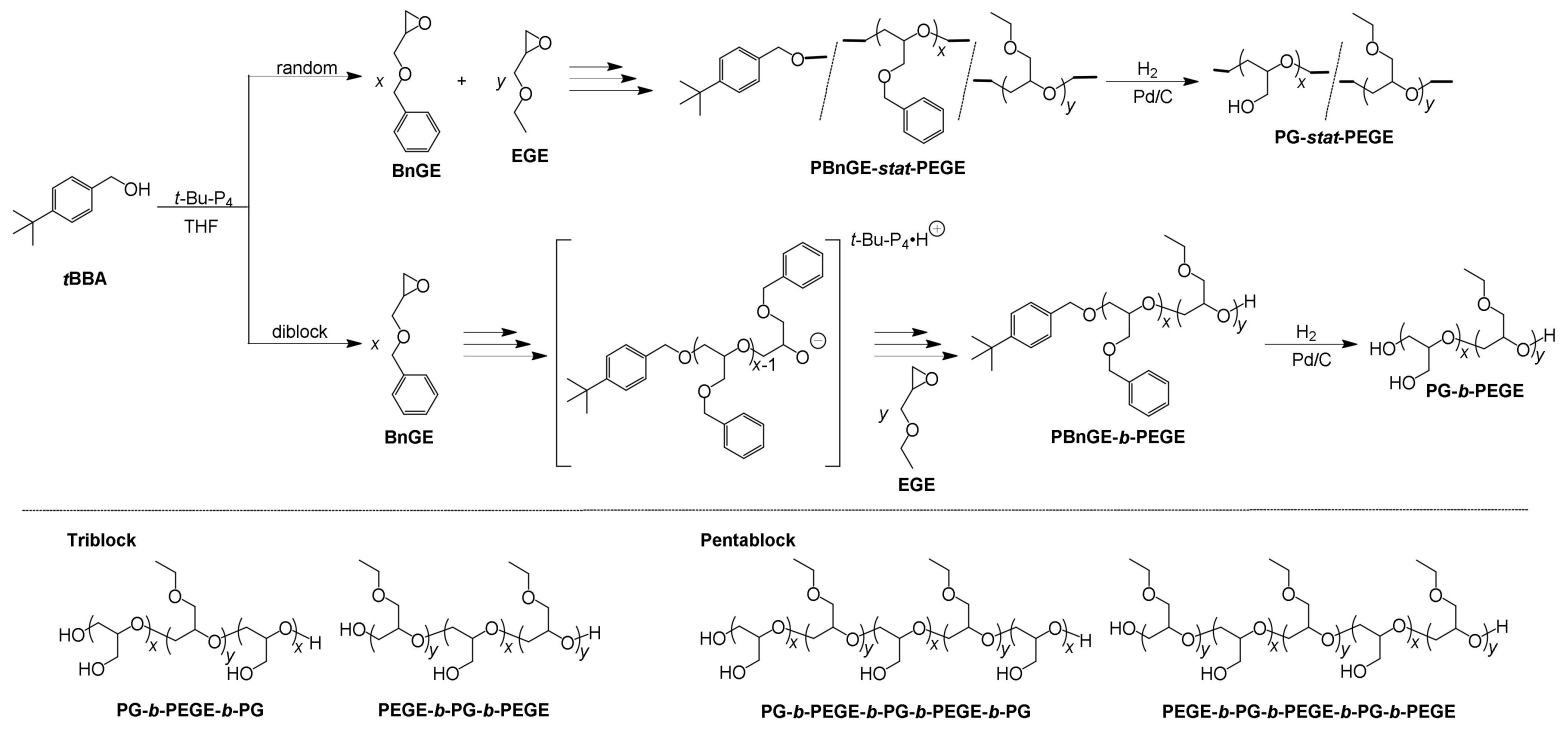
Synthesis of statistic and block copolymers (PG-*stat*-PEGE and PG-*b*-PEGE, PG-*b*-PEGE-*b*-PG, PEGE-*b*-PG-*b*-PEGE, PG-*b*-PEGE-*b*-PG-*b*-PEGE-*b*-PG, and PEGE-*b*-PG-*b*-PEGE-*b*-PG-*b*-PEGE, respectively).

In this study, we designed the copolymerization system of BnGE and EGE to extend to the synthesis of block copolymers and statistic copolymers without any specific end groups, that is, the multiple copolymers using PG as the hydrophilic chain segment and PEGE as the temperature-sensitive segment, as shown in Scheme 1. This study reports: (1) the living controlled ROP was catalyzed by *t*-Bu-P_4_ using *t*BBA as initiator through the statistic and block copolymerization methods and then deprotected to obtain PG_x_-*stat*-PEGE_y_ and PG_x_-*b*-PEGE_y_, and the thermoresponsive properties of these copolymers with different x/y ratios were comprehensively studied; (2) the synthesis of multiblock copolymers composed of PG and PEGE chains, PG_15_-*b*-PEGE_70_-*b*-PG_15_, PG_35_-*b*-PEGE_30_-*b*-PG_35_, PEGE_15_-*b*-PG_70_-*b*-PEGE_15_, PEGE_35_-*b*-PG_30_-*b*-PEGE_35_, PEGE_20_-*b*-PG_20_-*b*-PEGE_20_-*b*-PG_20_-*b*-PEGE_20_, and PG_20_-*b*-PEGE_20_-*b*-PG_20_-*b*-PEGE_20_-*b*-PG_20_, to explore the effect of the disorder to the copolymers on the thermoresponsive property; (3) we not only designed and synthesized the homopolymer PEGE without specific α-chain ends but also used dynamic light-scattering (DLS) measurement to explore the thermoresponsive properties of polymers in aqueous solutions.

## 2. Materials and Methods

### 2.1. Materials

Dichloromethane, extra dry, with molecular sieves (water <50 ppm) was purchased from Energy Chemicals Co., Inc. (Anhui, China) and was used as received. Dry toluene (purity, >99.5%, water content, <0.001%) for polymerization was purchased from Energy Chemicals Co., Ltd. and distilled in an Ar atmosphere over sodium benzophenone. Benzyl glycidyl ether (BnGE), 4-*tert*-butyl-benzyl alcohol (*t*BBA), and glycidyl ethyl ether (EGE) were purchased from Tokyo Kasei Kogyo Co., Ltd. (TCI, Tokyo, Japan) and distilled over CaH_2_ two times prior to use. 1-*tert*-Butyl-4,4,4-tris(dimethylamino)-2,2-bis[tris (dimethylamino) phosphoranylid enamino]-2Λ^5^,4Λ^5^-catenadi(phosphazene) (*t*-Bu-P_4_; 0.8 M solution in *n*-hexane) was purchased from Sigma-Aldrich (St. Louis, MO, USA). Palladium on charcoal (Pd/C, 10%) was purchased from Energy Chemicals Co., Ltd. and was used as received.

### 2.2. Synthesis of PBnGE-stat-PEGE

A typical procedure for the statistic copolymerization of BnGE and EGE is described as follows: To a solution of *t*BBA (6.57 mg, 40 μmol) in 1.41 mL dry toluene, 0.05 mL of *t*-Bu-P_4_ (40 μmol as 0.8 M solution in *n*-hexane), EGE (0.20 g, 2 mmol), and BnGE (0.33 g, 2 mmol) were added, in this order. The color of the solution changed from light yellow to dark yellow, indicating that the polymerization was successfully initiated. The polymerization was then quenched by adding a small amount of benzoic acid mixture to the polymerization solution. Aliquots were removed from the polymerization mixture to determine the conversion of BnGE and EGE by ^1^H NMR measurements. The obtained polymer was purified through neutralized aluminum oxide to obtain PBnGE_50_-*stat*-PEGE_50_ as a colorless and viscous liquid. Yield, 92.9%; *M*_n, NMR_, 13.5 kg mol^−1^; *M*_w_/*M*_n_, 1.07.

### 2.3. Synthesis of PG-stat-PEGE

A typical deprotection procedure is described as follows: Palladium on carbon (10% Pd/C; 100 mg) was added to a solution of PBnGE_50_-*stat*-PEGE_50_ (100 mg; 7.41 μmol) in a mixture of methanol and CH_2_Cl_2_ (1/1, *v*/*v*; 10.00 mL), and the whole mixture was stirred under a hydrogen atmosphere (3–5 MPa) for 24 h. After removing the Pd/C catalyst by filtration using celite, the solution was evaporated to obtain PG_50_-*stat*-PEGE_50_ as a colorless and viscous liquid. Yield, 93.4%; *M*_w,MALS_, 8.8 kg mol^−1^; *M*_w_/*M*_n_, 1.10.

### 2.4. Synthesis of PBnGE-b-PEGE

A typical procedure for the diblock copolymerization of BnGE and EGE is described as follows: To a solution of *t*BBA (6.57 mg, 40 μmol) in 1.41 mL dry toluene, 0.05 mL of *t*-Bu-P_4_ (40 μmol as 0.8 M solution in *n*-hexane) and BnGE (0.33 g, 2 mmol) were added, in this order. The color of the solution changed from light yellow to dark yellow, indicating that the polymerization was successfully initiated. After completion of BnGE polymerization (20 h, determined by the conversion of BnGE by ^1^H NMR measurements), EGE (0.20 g, 2 mmol) was further added to the polymerization mixture to continue the block copolymerization for 20 h. The polymerization was then quenched by adding a small amount of benzoic acid mixture to the polymerization solution. Aliquots were removed from the polymerization mixture to determine the conversion of BnGE and EGE by ^1^H NMR measurements. The obtained polymer was purified through neutralized aluminum oxide to obtain PBnGE_50_-*b*-PEGE_50_ as a colorless and viscous liquid. Yield, 93.6%; *M*_n, NMR_, 13.5 kg mol^−1^; *M*_w_/*M*_n_, 1.05.

### 2.5. Synthesis of PG-b-PEGE

A typical deprotection procedure is described as follows. Palladium on carbon (10% Pd/C; 100 mg) was added to a solution of PBnGE_50_-*b*-PEGE_50_ (100 mg; 7.41 μmol) in a mixture of methanol and CH_2_Cl_2_ (1/1, *v*/*v*; 10.00 mL), and the whole mixture was stirred under a hydrogen atmosphere (3–5 MPa) for 24 h. After removing the Pd/C catalyst by filtration using celite, the solution was evaporated to obtain PG_50_-*b*-PEGE_50_ as a colorless and viscous liquid. Yield, 90.5%; *M*_w,MALS_, 8.8 kg mol^−1^; *M*_w_/*M*_n_, 1.13.

### 2.6. Characterization

The ^1^H and ^13^C NMR spectra were recorded using a Bruker AVANCE III HD 500 (Bruker, Billerica, MA, USA) test in Changchun, China.

The polymerization solution was prepared in a MIKROUNA glove box (Changchun, China) equipped with a gas-purification system (molecular sieves and copper catalyst) and a dry argon atmosphere (H_2_O, O_2_ < 1 ppm). The moisture and oxygen contents in the glove box were monitored by a MK-XTR-100 and a MK-OX-SEN-1, respectively.

The number of average molecular weights (*M*_n_s) and molecular weight distributions (*M*_w_/*M*_n_s) of the polymers were measured by size exclusion chromatography (SEC) using an Agilent high-performance liquid chromatography (1260 Infinity II) system (Agilent, Santa Clara, CA, USA) in THF or H_2_O in the presence of 0.05% NaN_3_ at a flow rate of 1.0 mL min^−1^. The weight average molecular weight (*M*_w,MALS_) was determined by the SEC equipped with a multiangle laser light-scattering (MALS) instrument (Wyatt Technology, DAWN 8 HELEOS^®^) (Santa Barbara, CA, USA) in DMF in the presence of 0.01M LiCl at a flow rate of 1.0 mL min^−1^. SEC(THF): Agilent PLgel (7.5 × 300 mm, average bead size: 10 μm, exclusion limit: 2 × 10^6^ g mol^−1^), PLgel (7.5 × 300 mm, average bead size: 10 μm, exclusion limit: 4 × 10^8^ g mol^−1^), columns. SEC(DMF): Agilent Polar Gel-M (7.5 × 300 mm, average bead size: 5 μm, exclusion limit: 2 × 10^4^ g mol^−1^), Polar Gel-M (7.5 × 300 mm, average bead size: 5 μm, exclusion limit: 4 × 10^6^ g mol^−1^), columns.

The hydrodynamic diameter (*D*_h_s) of the obtained polymer was analyzed using a dynamic light-scattering (DLS) detector (Wyatt Technology, Dyna Pro Nanostar^®^) test in Changchun, China.

The cloud-point measurements were performed based on the ultraviolet-visible (UV-vis) spectra by passing through a 10-mm path-length cell using a Jasco V-770 spectrophotometer (Tokyo, CA, Japan) equipped with a temperature-controller (Jasco CTU-100) test in Changchun, China. The temperature was increased at the rate of 1 °C min^−1^, and the transmittance change with temperature was recorded using a spectrophotometer at the wavelength of 500 nm.

The matrix-assisted laser desorption/ionization time-of-flight mass spectrometry Bruker Autoflex III (MALDI-TOF MS) (Bruker, Billerica, MA, USA) measurements were performed using an Applied Biosystems Voyager-DE STR-H mass spectrometer with a 25-kV acceleration voltage test in Changchun, China. The positive ions were detected in reflector mode (25 kV). A nitrogen laser (337 nm, 3 ns pulse width, 106–107 W cm^−2^) operating at 3 Hz was used to produce the laser desorption, and the 200 shots were summed. The spectra were externally calibrated using a sample prepared from narrow-dispersed polystyrene. Samples for the MALDI-TOF MS were prepared by mixing the polymer (1.5 mg mL^−1^, 10 μL), the matrix (trans-3-indoleacrylic acid, 10 mg mL^−1^, 90 μL), and the actionizing agent (sodium trifluoroacetate, 10 mg mL^−1^, 10 μL) in THF.

## 3. Results and Discussion

### 3.1. Synthesis of Statistic Copolymers and Block Copolymers

**Figure 1:**
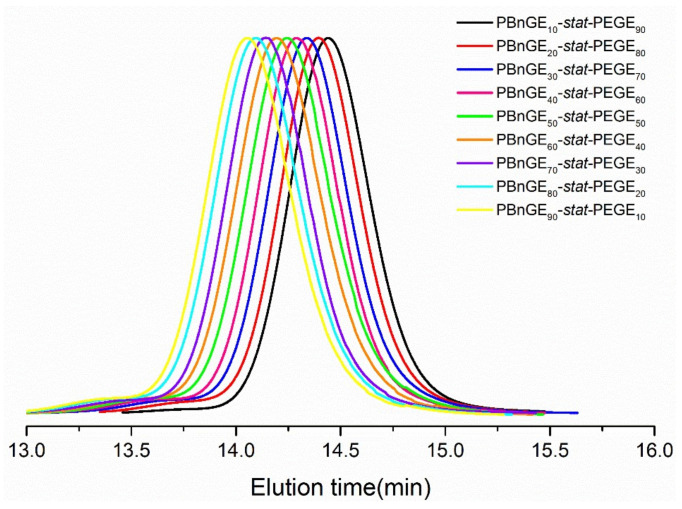
SEC trace of PBnGE_x_-*stat*-PEGE_y_ in THF.

For preliminary research, we studied a series of copolymers of BnGE and EGE obtained by statistic/block copolymerization methods, i.e., PBnGE-*stat*-PEGE, PBnGE-*b*-PEGE. In accordance with Scheme 1, PBnGE was first prepared by the *t*-Bu-P_4_-catalyzed anionic ROP of the monomer BnGE using 4-*tert*-butylbenzyl alcohol (*t*BBA) as the initiator, followed by EGE as the M_2nd_ monomer for statistic/block copolymerization. Table 1 shows the list of the copolymerization results. Here, the total number with the ratio of monomers to initiator ([M_1st_]_0_/[tBBA]) and ([M_2nd_]_0_/[*t*BBA]) in the two stages is controlled at 100 to produce the copolymers with different molecular weights. All polymerization reactions are carried out with the monomer conversion rate (conversion rate >99.9%) to produce the corresponding copolymers, which required molecular weight ranges from 10.8 to 15.6 kg mol^−1^. We prepared copolymers through the random copolymerization of BnGE with EGE, which are termed as, statistical copolymers. All of the size exclusion chromatography (SEC) traces of the PBnGE-*stat*-PEGE (Figure 1) showed a unimodal distribution, which demonstrated narrow dispersity (*M*_w_/*M*_n_) values of no more than 1.1. Obviously, as the ratio of [PBnGE]_0_/[*t*BBA]_0_ increased and [PEGE]_0_/[*t*BBA]_0_ decreased, it shifted to the higher molecular-weight region. The synthesis of PBnGE-*stat*-PEGE was carried out under the conditions of [PBnGE]_0_/[EGE]_0_= 10/90, 20/80, 30/70, 40/60, 50/50, 60/40, 70/30, 80/20, and 90/10 (entries 1–9). Similarly, the syntheses of PBnGE-*b*-PEGE were performed with [M_1st_]_0_/[M_2nd_]_0_=10/90, 20/80, 30/70, 40/60 and 50/50 (runs 10–14), respectively. In addition, the SEC trace showed a symmetrical monomodal peak with the *M*_w_/*M*_n_ value of 1.05–1.08, which implied the absence of the side reactions like chain transfer during the block.

**Figure 2:**
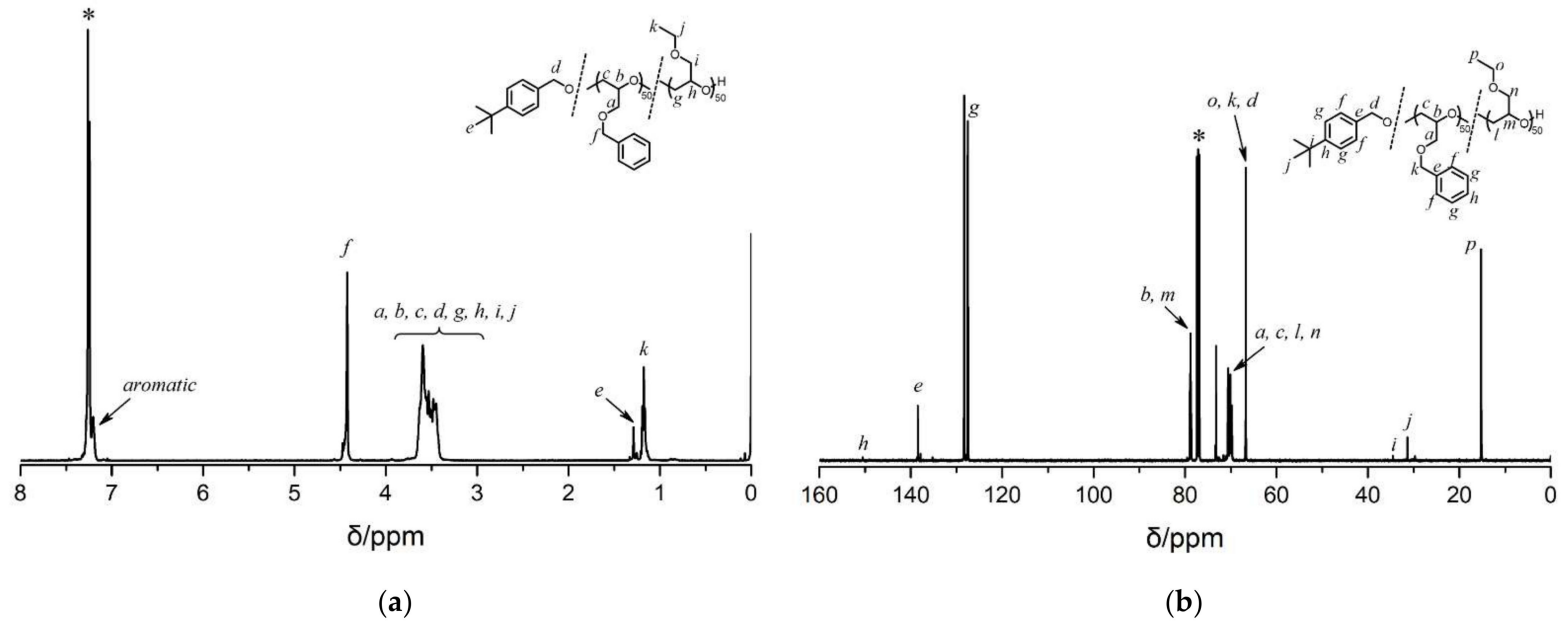
^1^H NMR (**a**) and ^13^C NMR (**b**) spectra of PBnGE_50_*-stat-*PEGE_50_ in CDCl_3_ (the symbol * refers to solvent peaks).

As shown in Figure 2, the structures of PBnGE_50_-*stat*-PEGE_50_ (entry 5, Table 1) were verified by the ^1^H NMR and ^13^C NMR spectra. The ^1^H NMR test revealed the comonomer ratio of BnGE to EGE and proved the presence of *t*BBA, which initiated the polymer. Comparing the integrals of the characteristic ethyl group (*f*) peaks of BnGE at 4.45 ppm and the characteristic methyl group (*k*) peaks of EGE at 1.16 ppm revealed results very consistent with the comonomer ratio, with the targeted 1 to 1 ratio. The characteristic allyl (*e*) peaks at 1.36 ppm, assigned to the *tert*-butyl group at the α-chain end in PBnGE-*stat*-PEGE, confirmed that the presence of *t*BBA initiated the copolymerization. The peaks of the methylene protons (*a*, *c*, *g*, and *i*) and the methine protons (*b* and *h*) in the polymer main chains at 3.38–3.74 ppm, as well as those assigned to the methylene group protons (*j*) of EGE peak and the methylene proton (*d*) peaks of *t*BBA, obviously verified the successful copolymerization of PBnGE-*stat*-PEGE. Combined NMR and SEC measurements revealed the final composition of the statistic copolymer as PBnGE-*stat*-PEGE. Technically, ^1^H NMR measurements still provided reliable information on the average degree of polymerization of the units generated for BnGE and EGE polymerizations (x and y, respectively). Additionally, the ^1^H NMR spectra can obtain the number average molecular weights (*M*_n,NMRs_) of the copolymer, which is consistent with the calculated molecular weight (*M*_n,calcd_) determined by the monomer conversion and even the [M_1st_]_0_/[M_2nd_]_0_/[ROH]_0_ values. Representative examples are as follows: the *M*_n,NMR_ (*M*_n,calcd_) values are 10.8 (11.0), 11.5 (11.6), 12.2 (12.2), 12.9 (12.9), and 13.5 (13.5) kg mol^−1^ of PBnGE-*stat*-PEGE (Table 1, entries 1–5). In summary, PBnGE_10_-*stat*-PEGE_90_, PBnGE_20_*-stat-*PEGE_80_, PBnGE_30_*-stat-*PEGE_70_, PBnGE_40_*-stat-*PEGE_60_, PBnGE_50_*-stat-*PEGE_50_, PBnGE_60_*-stat-*PEGE_40_, PBnGE_70_*-stat-*PEGE_30_, PBnGE_80_*-stat-*PEGE_20_, and PBnGE_90_*-stat-*PEGE_10_ were synthesized, thus allowing debenzylation research in the next step.

**Figure 3:**
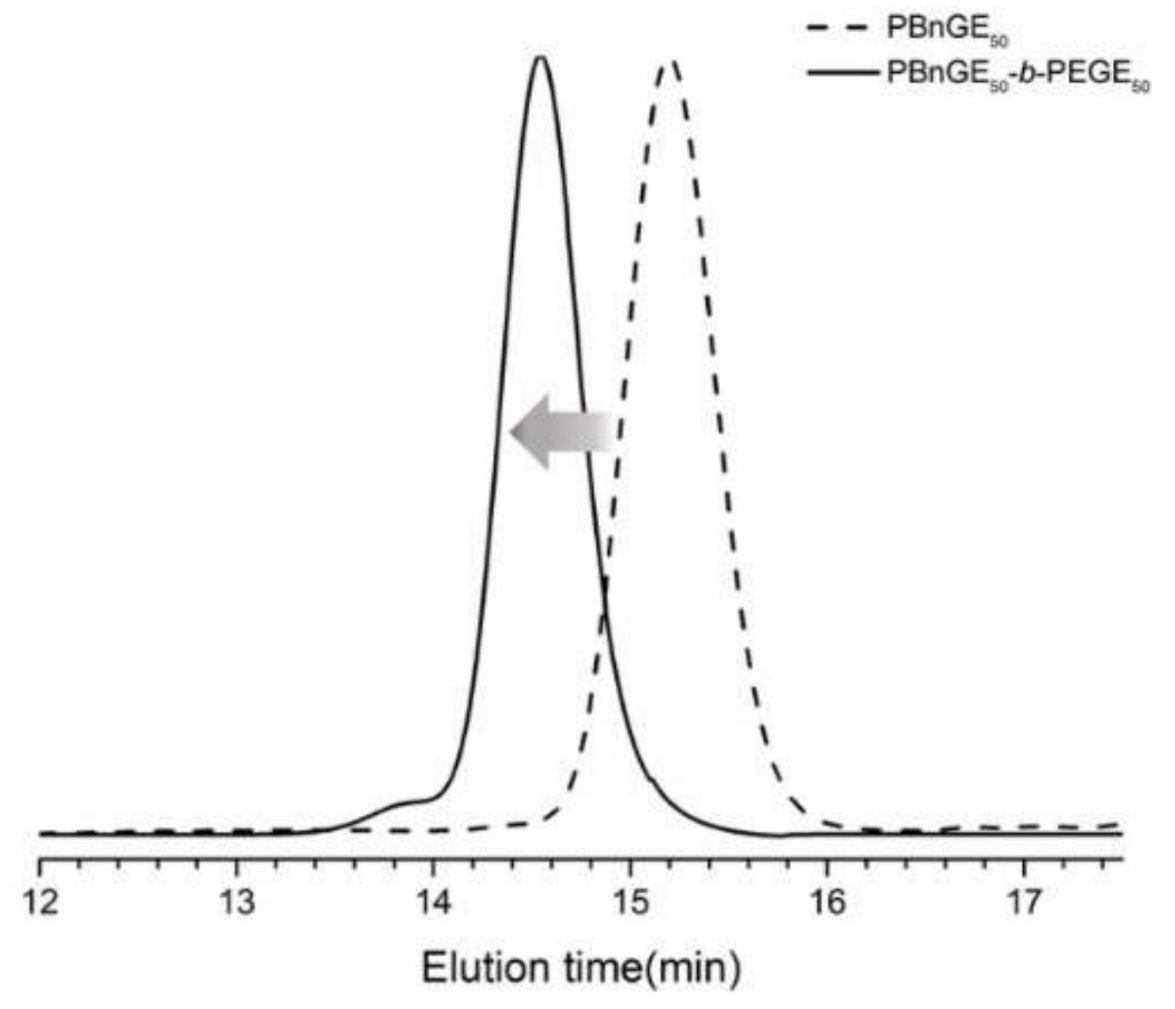
SEC traces of PBnGE_50_-*b*-PEGE_50_ in THF.

We aimed to investigate the thermoresponsive behaviors of the PG-*b*-PEGE consisting of PG and PEGE in order to gain insight into the effect of the monomer sequence. Thus, we conducted the sequential block copolymerization of PBnGE, followed by EGE, using the *t*-Bu-P_4_/*t*BBA system with [BnGE]_0_/[EGE]_0_/[*t*BBA]_0_ ratios of 10/90/1, 20/80/1, 30/70/1, 40/60/1, and 50/50/1 to afford a series of PBnGE_x_-*b*-PEGE_y_ with various x and y values (Scheme 1 and Table 1). As the first step of block copolymerization, *t*-Bu-P_4_ with a [BnGE]_0_/[*t*BBA]_0_/[*t*-Bu-P_4_]_0_ ratio of 50/1/1 catalyzed ROP of BnGE for 20 h. ^1^H NMR and SEC analysis of aliquots of the polymer showed that the monomer conversion rate reached 99%, and it was verified that active PBnGE oxyanions were formed, with an *M*_n, NMR_ value of 8.3 kg mol^−1^ and *M*_w_/*M*_n_ of 1.04. Then, the block copolymerization second stage was started by adding 50 equivalents of EGE relative to the *t*BBA used for the first polymerization. While monitoring the polymerization by ^1^H NMR, the quantitative consumption of EGE after 24 h can be observed. What is more, the ^1^H NMR and ^13^C NMR of the obtained copolymer PBnGE_50_-*b*-PEGE_50_ can capture the proton signal and carbon atom signal corresponding to PBnGE and PEGE, with calculated *M*_w_/*M*_n_ and *M*_n, NMR_ of 1.05 and 13.5 kg mol^−1^, respectively. As shown in Figure 3, the SEC trace of the final product, PBnGE_50_-*b*-PEGE_50_, has shifted to a higher molecular-weight region compared with the PBnGE_50_ obtained in the first stage of polymerization. It is not difficult to find that there is a small shoulder in the SEC curve in the higher molecular-weight region, but the *M*_w_/*M*_n_ value of 1.05, including the shoulder, is still very narrow. The only shoulder showed in this trace is in the high molecular-weight region, which could be from the termination of the polymerization. These results confirmed the successful block copolymerization to produce PBnGE_50_-*b*-PEGE_50_ with a predictable molecular weight and narrower *M*_w_/*M*_n_. PBnGE_10_-*b*-PEGE_90_, PBnGE_20_-*b*-PEGE_80_, PBnGE_30_-*b*-PEGE_70_, and PBnGE_40_-*b*-PEGE_60_ were also successfully prepared in the same manner. In addition, we also prepared the triblock and pentablock polymerizations (Table S1 and Table S3, Supplementary Material).

### 3.2. Synthesis and Characterizations of PG-stat-PEGE and PG-b-PEGE

In order to obtain the designed copolymers PG*-stat-*PEGE and PG-*b*-PEGE, we used Pd/C as the catalyst to hydrogenate and crack a total of 14 samples of PBnGE*-stat-*PEGE and PBnGE-*b*-PEGE, and further removed the benzyl (Bn) group and 4-*tert*-butylbenzyl (*t*BBn) in the PBnGE segment. The deprotection reaction was performed under a hydrogen atmosphere, and methanol/CH_2_Cl_2_ (1:1) was used as a mixed solvent at room temperature. The ^1^H NMR measurement was performed to further confirm the quantitative progress of each reaction, and then the samples were purified to obtain pure deprotected polymers in a viscous liquid state (entries 15–28 in Table 2). The SEC traces of the PG*-stat-*PEGE after the debenzylation reaction are monomodal peaks, as shown in Figure S1 (Supplementary Material), the *M*_w_/*M*_n_ values of which are summarized in Table 2, ranging from 1.10 to 1.15, with a separation yield ranging from 85.3% to 93.5%. More importantly, by analyzing the values of *M*_w_/*M*_n_ in Table 1 and Table 2, it is concluded that the debenzylation reaction of the polymer did not lead to a significant broadening of the molecular-weight distribution.

**Figure 4:**
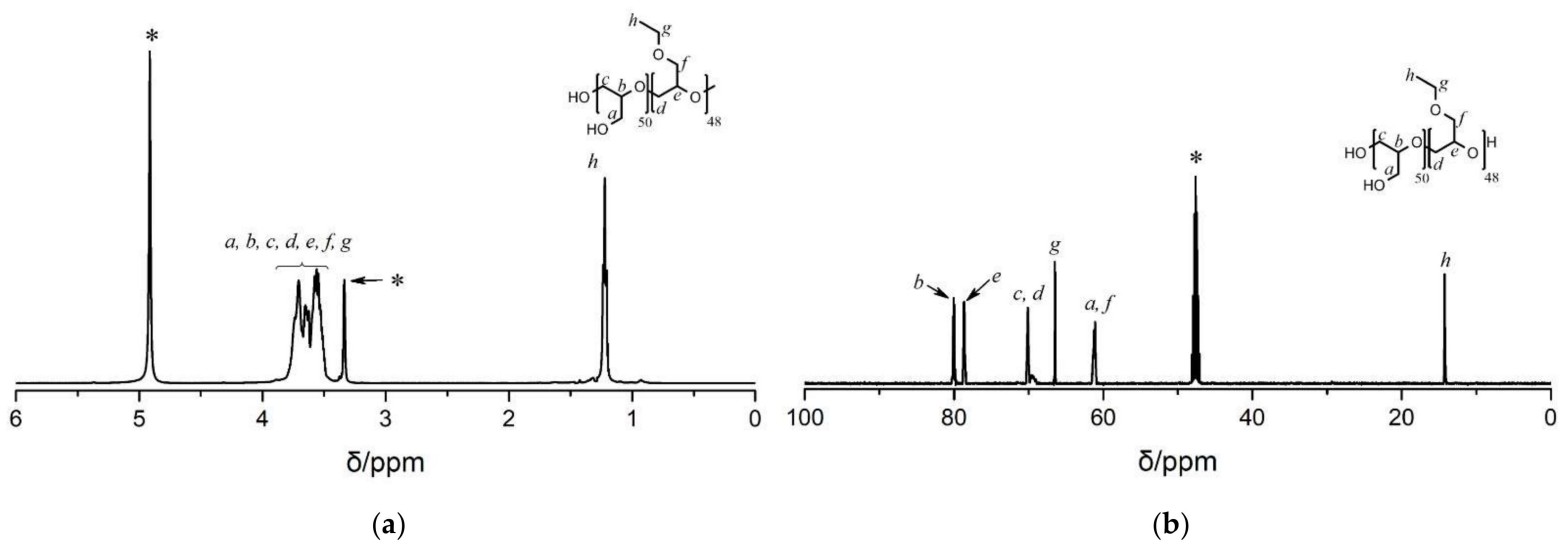
Typical ^1^H NMR (**a**) and ^13^C NMR (**b**) spectra of PG_50_*-b-*PEGE_50_ in MeOD (the symbol * refers to solvent peaks).

As shown in Figure 4, for the ^1^H NMR spectrum and the ^13^C NMR spectrum of PG_50_*-stat-*PEGE_50_, PG as the main chain segment protons (*a*, *b*, and *c*) appeared at 3.48–3.82 ppm, and the characteristic peaks of Bn and *t*BBn groups were not observed in the spectrum. However, the PG signals through the ^13^C NMR spectrum can be confirmed again, which appeared at 62.0, 80.4, and 70.8 ppm (*a*, *b*, and *c*, respectively). It is worth noting that although the characteristic peaks of the PEGE segment did not directly show in ^1^H NMR, the peaks, *g* and *h*, of the ethyl group, respectively, appeared at 66.5 and 14.6 ppm in ^13^C NMR. However, the above results indicated that deprotection quantitatively occurred in order to provide the copolymer PG*-stat-*PEGE without substituents at the α-end. Additionally, we used SEC equipped with a multiangle laser light scattering (SEC-MALS) instrument to test their actual weighted average molecular weights (M_w,MALS’s_), and the results for PG*-stat-*PEGE and PG-*b*-PEGE ranged from 7.2 to 9.7 kg mol^−1^ (entries 15–28 in Table 2). It is worth noting that as the α-end of copolymers are OH groups without characteristic peaks, we adopted the SEC measurements equipped with multi-angle laser light-scattering (SEC-MALLS) instruments to provide their weight average molecular weights (*M*_w, MALS_’s). As shown in Table 2, the actual weight average molecular weight (*M*_w, MALS_) of the copolymers PG_x_*-stat-*PEGE_y_ and PG_x_-*b*-PEGE_y_ is in the range of 7.4–9.7 kg mol^−1^.

### 3.3. Thermoresponsive Properties of PG_x_-stat-PEGE_y_ and PG_x_-b-PEGE_y_

**Figure 5:**
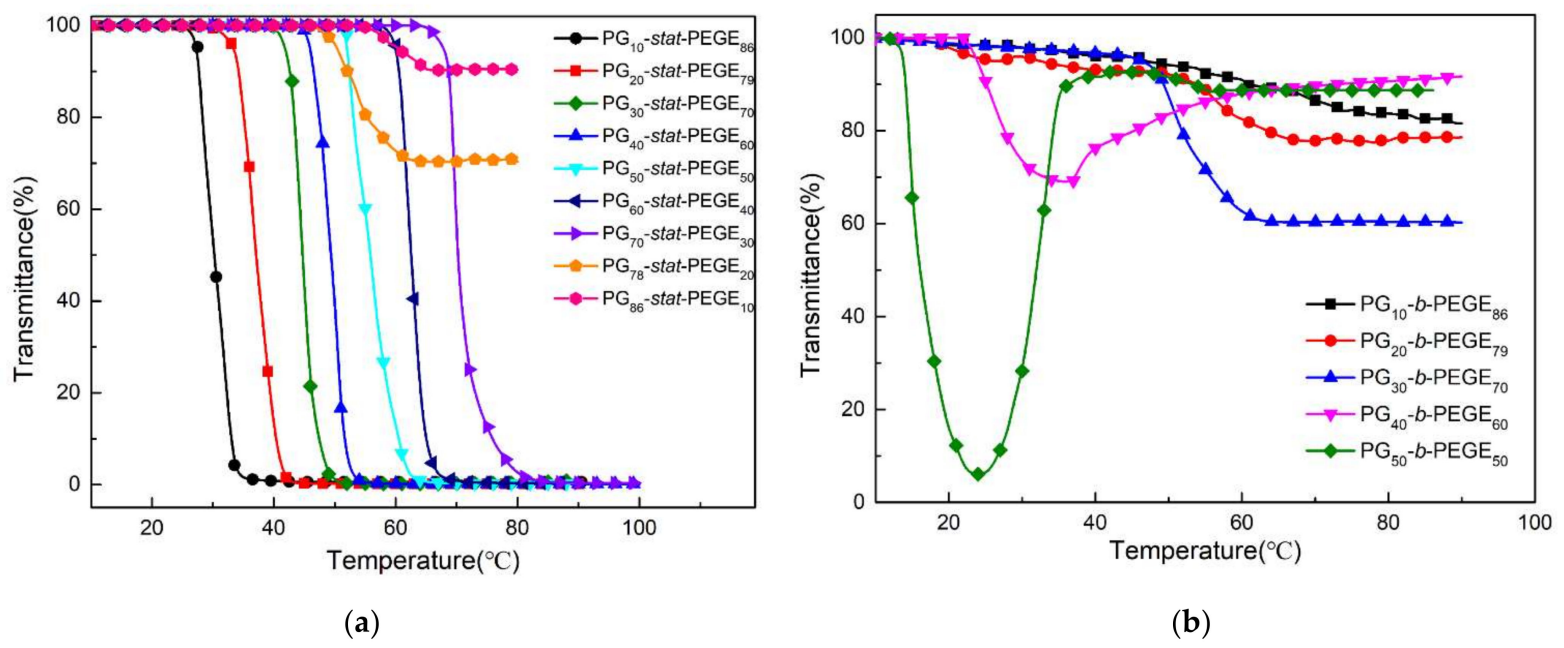
The cloud-point curves of (**a**) PG*_x_-stat-*PEGE*_y_* and (**b**) PG*_x_*-*b-*PEGE*_y_* in the aqueous solutions (10 g L^−1^).

The thermal phase-transition behavior of copolymers was determined by the transmittance-vs-temperature curves; that is, the *T*_cp_ was defined as the temperature when the transmittance reached 50%. The transmittance-vs-temperature curves are formed by monitoring the polymer solution at a concentration of 10 g L^−1^ with an ultraviolet-visible spectrophotometer at a wavelength of 500 nm. Figure 5 shows the dependence of the transmittance of PG*-stat-*PEGE and PG-*b*-PEGE on temperature. Among the obtained copolymers, both PG*-stat-*PEGE and PG-*b*-PEGE were soluble in pure water at room temperature. It was found that the aqueous solution of PG_10_*-stat-*PEGE_86_, PG_20_*-stat-*PEGE_79_, PG_30_*-stat-*PEGE_70_, PG_40_*-stat-*PEGE_60_, PG_50_*-stat-*PEGE_50_, PG_60_*-stat-*PEGE_40_, and PG_70_*-stat-*PEGE_30_ showed a sharp drop in transmittance when heated, indicating the existence of an T-type phase transition. In order to confirm the reversibility of the phase transition, we found that the optical transmittance of the solution returned to 100% after cooling, and the hysteresis was negligible. On the other hand, although PG_78_*-stat-*PEGE_20_ and PG_87_*-stat-*PEGE_10_ are completely soluble in water, the transmittance slightly decreases with increasing temperature. Surprisingly, it does not show any significant phase change at 90 °C. The aqueous solution of PG*_x_-stat-*PEGE*_y_* (x = 10, 20, 30, 40, 50, 60, and 70; y = 30, 40, 50, 60, 70, 79, and 86) reacts violently to thermal stimuli, as shown in Figure 5a, and the *T*_cp_ are listed in Table 2. For PG*-stat-*PEGE, we observed that *T*_cp_ increased with an increasing hydrophilic segment PG and a decreasing thermoresponsive segment PEGE, accompanied by decreasing of *M*_w, MALS_, which is usually observed in most thermoresponsive polymers. In other words, an earlier reduction in optical transmittance can be observed for PG*_x_-stat-*PEGE*_y_* with a higher EGE ratio, which is in line with our expectations. When the value of PG unit x increased to 78 and the value of PEGE unit y dropped to 20, it was difficult to exhibit LCST phase-transition behavior.

**Figure 6:**
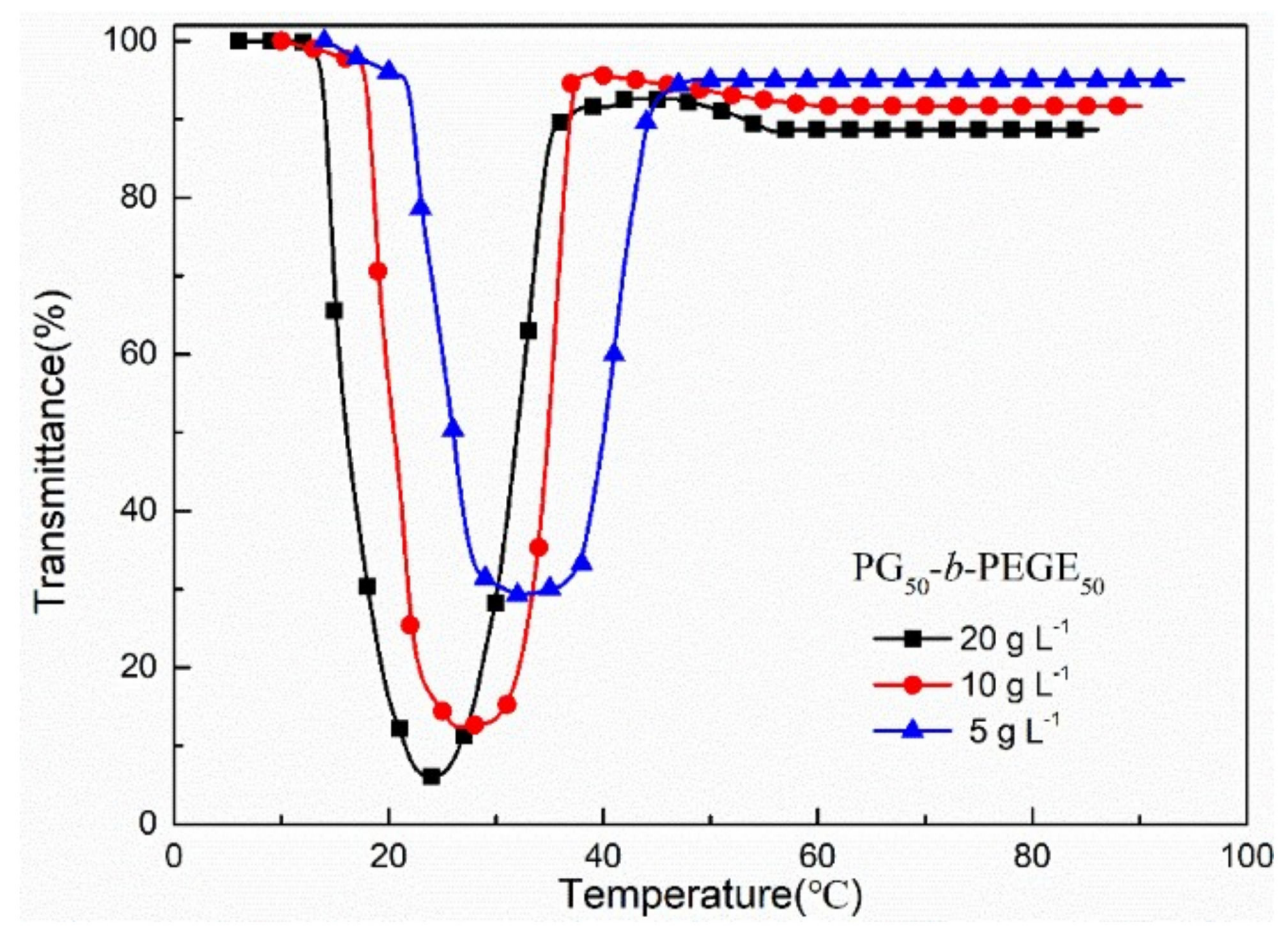
The thermal phase-transition curves at different concentrations of PG_50_-*b-*PEGE_50_.

Different from PG*-stat-*PEGE’s performance, PG_10_-*b*-PEGE_86_, PG_20_-*b*-PEGE_79_, and PG_30_-*b*-PEGE_70_ did not show a sharp drop in transmittance from 0 °C to 90 °C, only slightly changing in transmittance. Interestingly, the transmittance of PG_40_-*b*-PEGE_60_ and PG_50_-*b*-PEGE_50_ in the range of 0–60 °C appears to first decrease with increased temperature and then rise to a high value after further heating, as shown in Figure 5b. Similar results have occurred when we can decompose this special phase-change behavior into first LCST-type and then temperature (UCST)-like (actually not UCST) phase transitions. In summary, the PG*_x_*-*b-*PEGE*_y_* phase transition significantly differs from that observed for PG*_x_-stat-*PEGE*_y_* when the values of x and y are the same. In addition, taking the thermoresponsive property characteristics of PG_50_-*b*-PEGE_50_ as an example, as shown in Figure 6, it can be concluded that the concentration of the polymer aqueous solution significantly affects the phase-transition process. In addition, the phase change becomes more obvious with increasing concentration, which may be attributed to the higher concentration and easier-to-form large aggregates. Therefore, in order to more directly understand the aggregation behavior of the polymer solution, we performed DLS measurements.

**Figure 7:**
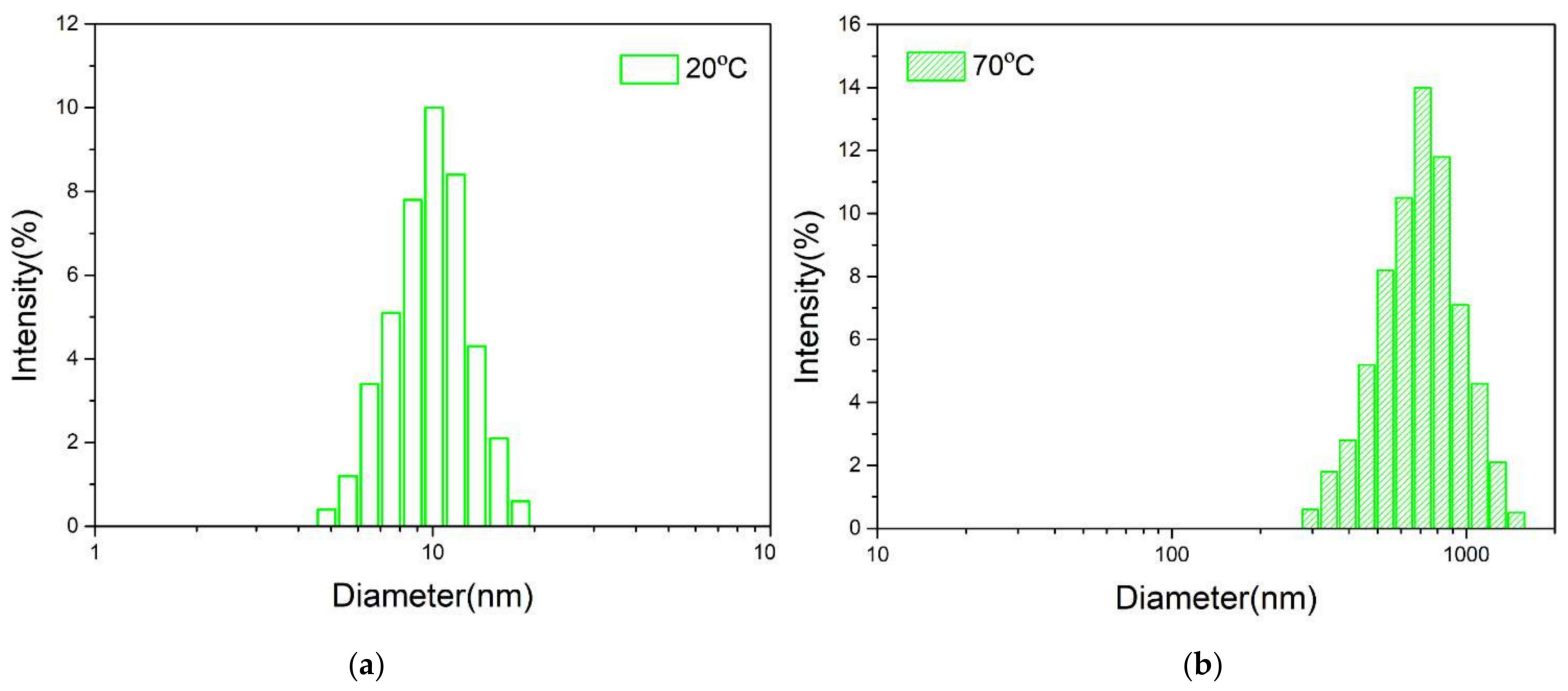
Hydrodynamic diameter distribution of PG*_50_-stat-*PEGE*_50_* at (**a**) 20 °C, and (**b**) 70 °C (scattering angle, 90°).

The hydrodynamic-diameter *(D*_h_) results of PG*_x_-stat-*PEGE*_y_* and PG*_x_*-*b-*PEGE*_y_* are summarized in Table 2. As an example, PG_50_*-stat-*PEGE_50_ was observed with a monodispersed distribution, and the *D*_h_ was 10.4 at 20 °C, as shown in Figure 7a. This value conforms to the order of a single polymer chain, which proves that there is only a single polymer chain in the solution. When at a high temperature of 70 °C, the DLS measurement curve of PG_50_*-stat-*PEGE_50_ still shows the monodispersed scattering distribution, whose *D*_h_ value calculated from the peak is 722 nm, and it is used as a typical distribution of PG*_x_-stat-*PEGE*_y_* in Figure 7b. Obviously, the *D*_h_ results show that PG_50_*-stat-*PEGE_50_ forms uniform aggregates with larger particle sizes at a higher temperature than *T*_cp_, resulting in a polymer aqueous solution with a permeability of 0. For PG_50_-*b*-PEGE_50_, there is a two-stage phase transition during the heating process; that is, the average measured value of *D*_h_ is only 9.6 nm at 5 °C. It rose to 268 nm quickly when the temperature increased to 25 °C. The *D*_h_ then undergoes a second transition to 26 nm due to heating to 40 °C. We consider that there may be a micelle-like structure at 10 °C, so in the first stage of raising the temperature, the micelles aggregate to form large aggregates with a uniform *D*_h_ of 268 nm. After the second stage of heating to above 40 °C, *D*_h_ decreased to 40 nm, and the aggregates rearranged to form more stable, tiny aggregates. In other words, the solution is in a metastable system state at 25 °C, and a phase transition from the first stage to the second stage can occur when the temperature reaches 45 °C. Comparing the phase-transition behavior of PG*_x_-stat-*PEGE*_y_* and PG*_x_*-*b-*PEGE*_y_*, we infer that the phase-transition behavior is completely different due to the disorder of the polymer chain. In order to demonstrate the point of disorder, we designed the synthesis of multiblock copolymers.

### 3.4. Thermoresponsive Properties of Multiblock Copolymer and Homopolymer

**Figure 8:**
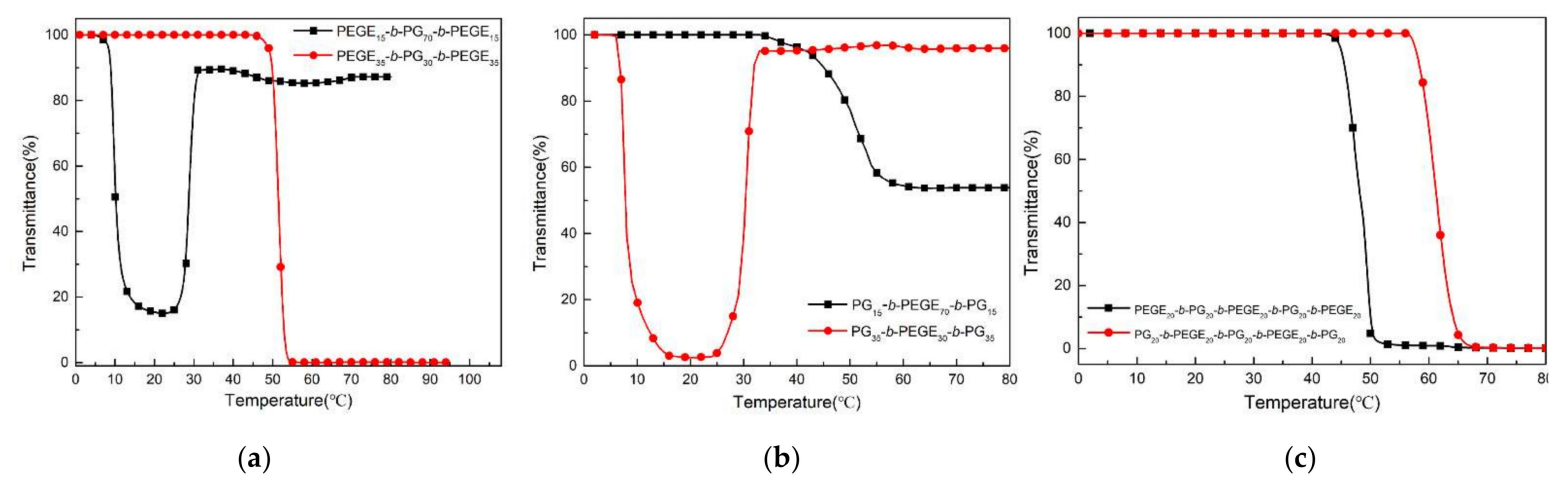
The thermal phase-transition curves at different concentrations of (**a**) PEGE_15_-*b*-PG_70_-*b-*PEGE_15_ and PEGE_35_-*b*-PG_30_-*b-*PEGE_35_, (**b**) PG_15_-*b*-PEGE*_70_*-*b-*PG_15_ and PG_35_-*b*-PEGE_30_-*b-*PG_35_, (**c**) PEGE_20_-*b*-PG_20_-*b-*PEGE_20_-*b*-PG_20_-*b*-PEGE_20_ and PG_20_-*b*-PEGE_20_-*b-*PG_20_-*b*-PEGE_20_-*b*-PG_20_ in 10 g L^−1^.

In order to evaluate the thermoresponsive properties of multiblock polymers, we designed and synthesized four triblock copolymers, PG_15_-*b*-PEGE_70_-*b*-PG_15_, PG_35_-*b*-PEGE_30_-*b*-PG_35_, PEGE_15_-*b*-PG_70_-*b*-PEGE_15_, and PEGE_35_-*b*-PG_30_-*b*-PEGE_35_. As shown in Figure 8a,b, our analysis shows that the thermal-response behavior of these four triblock copolymers significantly depends on the ratio of PG/PEGE. As observed, PEGE_35_-*b*-PG_30_-*b*-PEGE_35_ showed a typical LCST-type phase transition, and PG_15_-*b*-PEGE_70_-*b*-PG_15_ and PEGE_15_-*b*-PG_70_-*b*-PEGE_15_ showed a two-stage phase transition. However, PG_35_-*b*-PEGE_30_-*b*-PG_35_ showed little change in transmittance. PEGE_35_-*b*-PG_30_-*b*-PEGE_35_ shows a typical LCST-type phase transition with a *T*_cp_ of 49.8 °C (Table S2). The thermal-response behaviors of PG_35_-*b*-PEGE_30_-*b*-PG_35_ and PEGE_15_-*b*-PG_70_-*b*-PEGE_15_ are very similar to the diblock copolymer of PG_50_-*b*-PEGE_50_ (Figure 5b, Table 2), in which transmittance reached close to 0% at the inflection point of the phase change. In addition, although the transmittance of PG_15_-*b*-PEGE_70_-*b*-PG_15_ decreased after the temperature was raised to 30 °C, the transmittance no longer decreased when it reached 55% at 50 °C. The *T*_cp_ of PEGE_20_-*b*-PG_20_-*b*-PEGE_20_-*b*-PG_20_-*b*-PEGE_20_ and PG_20_-*b*-PEGE_20_-*b*-PG_20_-*b*-PEGE_20_-*b*-PG_20_ results shown in Figure 8c were 48.3 and 61.7 °C, respectively. In addition, although their *M*_w,MALS_ are similar to each other in Table S4, it is not difficult to find that the higher percentage of EGE units tends to have lower *T*_cp_. It should be noted that the pentablock copolymers PEGE_20_-*b*-PG_20_-*b*-PEGE_20_-*b*-PG_20_-*b*-PEGE_20_ and PG_20_-*b*-PEGE_20_-*b*-PG_20_-*b*-PEGE_20_-*b*-PG_20_ both exhibited LCST-type phase transitions in aqueous solutions, which were in sharp contrast to the results of triblock and diblock copolymers. Finally, we confirmed our conjecture that the dependence of *T*_cp_ on the disorder of copolymers is undeniable. However, the exact of cause this phenomenon is unclear.

As one example, Figure S2 shows the ^1^H NMR spectra of PEGE_15_-*b*-PG_70_-*b*-PEGE_15_ with the temperature changed in D_2_O. Since the instrument cannot cool down at room temperature, the temperatures were initially set to 23 °C, increased to 33 °C and 43 °C, then finally cooled to 23 °C. For the initial 23 °C measurement, the signals of the main chains and side groups could be clearly observed at 3.81–3.22 ppm (a, b, c, d). The signals obviously shifted to the low magnetic-field regions when the temperature increased from 23 °C to 43 °C. Similar tendencies were observed for the other signals in the range of 1.03–1.27 ppm (e). This result can be explained by the theory that the electron densities of the protons decrease due to intramolecular/intermolecular bond interactions, resulting in the shift of the signals to the low magnetic-field regions. Notably, the signals were not significantly broadened but significantly enhanced by the increasing temperature. We cannot provide a reliable explanation for this unexpected result, but the enhancement of the signal corresponds to this increase in transmittance behavior. For the final 23 °C measurements, the spectral profile agreed with that of the initial one, proving that the phase-transition phenomenon is reversible. In order to further determine the interesting phase-transition behavior, we performed DLS measurements on these multiblock polymers to understand the polymer at each stage of the phase-transition hydrodynamic diameter (*D*_h_) at 20 and 60 °C. At high temperatures above 60 °C, DLS measurements show a monodispersed scattering distribution, which is a typical distribution curve for PG_15_-*b*-PEGE_70_-*b-*PG_15_ (Figure S3). For PG_15_-*b*-PEGE_70_-*b-*PG_15_, the *D*_h_ at 60 °C is calculated as 545 nm. The results showed that although the light transmittance did not reach 0%, PG_15_-*b*-PEGE_70_-*b-*PG_15_ had formed stable and uniform aggregates upon *T*_cp_ attributed to the formed aggregates were not observed to aggregate further. In addition, the *D*_h_ values of PEGE_15_-*b*-PG_70_-*b-*PEGE_15_, PEGE_35_-*b*-PG_30_-*b-*PEGE_35_, PG_35_-*b*-PEGE_30_-*b-*PG_35_, PEGE_20_-*b*-PG_20_-*b-*PEGE_20_-*b*-PG_20_-*b*-PEGE_20_, and PG_20_-*b*-PEGE_20_-*b-*PG_20_-*b*-PEGE_20_-*b*-PG_20_ are shown in Table S4.

**Figure 9:**
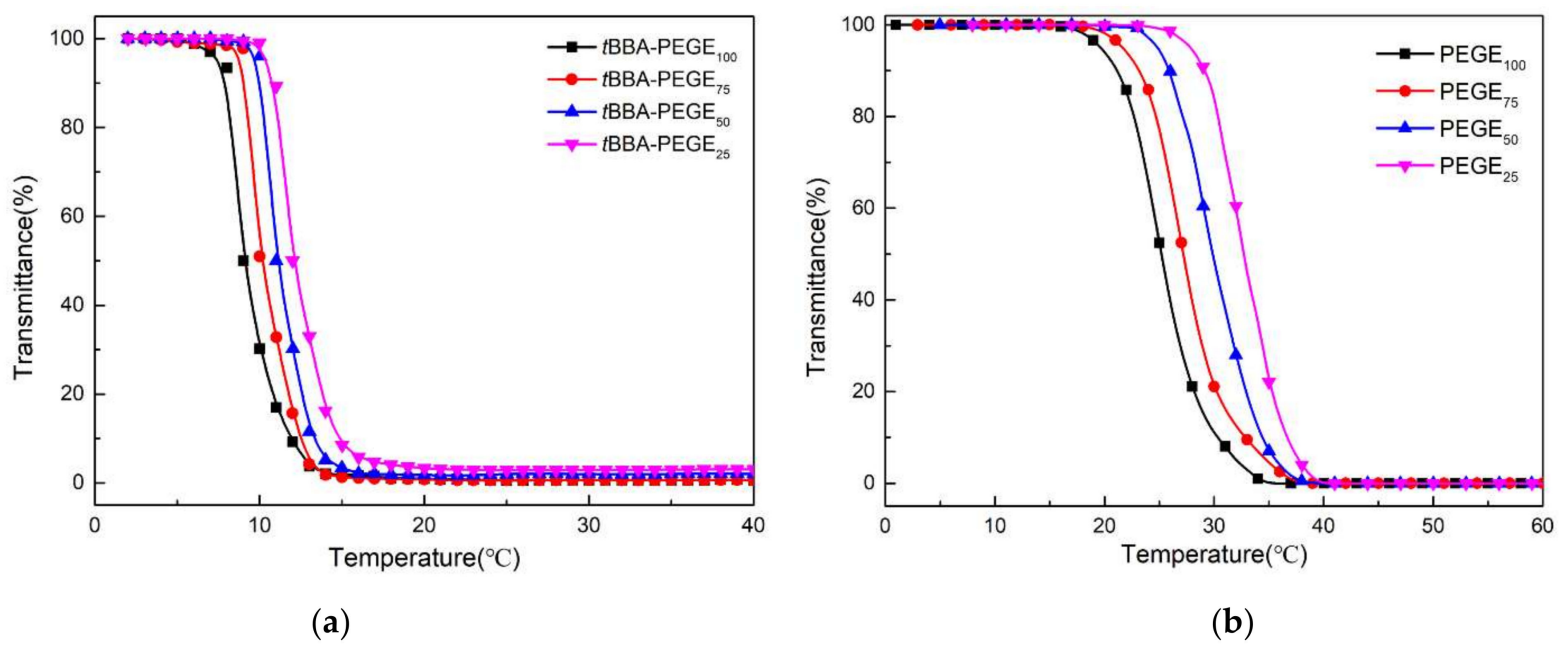
The thermal phase-transition curves at different concentrations of (**a**) *t*BBA-PEGE_25_, *t*BBA-PEGE_50_, *t*BBA-PEGE_75_, and *t*BBA-PEGE_100_ and (**b**) PEGE_25_, PEGE_50_, PEGE_75_, and PEGE_100_.

We considered that while exploring the thermoresponsive property of the copolymer of PG and PEGE, it should be necessary to explore the homopolymer of PEGE to compare. Our group reported the synthesis of PEGE with the *n-*butoxy group as the α-chain end in previous studies [34]. However, we first used *t*BBA as an initiator to carry out ring-opening polymerization to obtain *t*BBA-PEGE in order to obtain the PEGE with a hydroxyl group at the α-chain end. Here, we designed the ratio of the initial monomer to the initiator ([M]_0_/[*t*BBA]) to be 25, 50, 75, and 100 to produce *t*BBA-PEGE with different molecular weights, which is obtained with a corresponding molecular weight of 2.7–10.4 kg mol^−1^ (Table S5). In addition, by observing the signal displayed by the ^1^H NMR spectrum, we can confirm that the resulting product affords a *t*BBA group at the end of the α-chain (Figure S4a). For the ^13^C NMR spectrum (Figure S4b), not only did the three signals appear at 61.0, 79.5, and 69.4 ppm (a, b, and c, respectively), but the signals of *t*BBA can also be observed. Figure S5 shows the matrix-assisted laser-desorption ionization time-of-flight mass spectrometry (MALDI-TOF MS) spectrum, which provides favorable evidence for the structure assignment of *t*BBA-PEGE. It can be seen from Figure 9a that the aqueous solutions of *t*BBA-PEGE_25, 50, 75, 100_ all become opaque during heating, indicating the existence of the LCST-type phase transition. As listed in Table S5, the *T*_cp_s of the polyethers were observed in the temperature range of 9.1–12.1 °C and increased in the following order: *t*BBA-PEGE_100_ (9.1 °C) < *t*BBA-PEGE_75_ (10.4 °C) < *t*BBA-PEGE_50_ (11.5 °C) < *t*BBA-PEGE_25_ (12.1 °C).

We synthesized the polymer PEGE with the α-chain end as the hydroxyl group through deprotection experiments and through a ^1^H NMR spectrum test to confirm that the *t*BBA group of the previous α-chain end had disappeared. In Figure S6, two signals appeared at 1.23–1.42 ppm and 3.48–3.85 ppm (*e* and *a*, *b, c, d,* respectively). The size exclusion chromatography (SEC) experiment (Figure S7) with the obtained products, PEGE, showed that the narrow dispersions (*M*_w_/*M*_n_) were less than 1.10. However, these results indicated that we successfully afforded PEGE without substituents in both ends of the chain after deprotection. The aqueous solutions of PEGE (10 g L^−1^) were characterized by UV-Vis spectroscopy to determine their *T*_cp_s, which are listed in Table S6. The *T*_cp_ value of PEGE_25, 50, 75, 100_ was in the range of 25.2 to 32.5 °C, as shown in Figure 9b. It is easy to find that both *t*BBA-PEGE and PEGE follow the same rule that *T*_cp_ will decrease as the degree of polymerization increases. The exciting phenomenon is that PEGE with the same degree of polymerization has a higher *T*_cp_ value than *t*BBA-PEGE, so we consider the α-end group has a considerable influence on the thermoresponsive property of the polymer. We further performed the DLS measurement of PEGE to monitor the *D*_h_ at different temperatures. Figure S8 depicts the curve of hydrodynamic diameter (*D*_h_) before and after the phase transition of PEGE_50_. Thus, we concluded that the thermal-response behavior of *t*BBA-PEGE and PEGE made our research on the thermoresponsive properties of its copolymers more colorful.

## 4. Conclusions

The ROP, using the combination of *t*-Bu-P_4_ and the *t*BBA initiator, was efficient for synthesizing PBnGE*-stat-*PEGE and PBnGE-*b*-PEGE in different DPs with the intended molecular weights and relatively narrow molecular weight distributions, the controlled/living nature of could also afford PG*-stat-*PEGE and PG-*b*-PEGE. PG_x_*-stat-*PEGE_y_ exhibited LCST-type thermal-response behavior, and adjusting the x/y value caused a cloud-point temperature (*T*_cp_) in the range of 30.5 to 70.4 °C, which may expand its application possibilities. To further study the thermal-response behavior of its copolymers, we synthesized triblock and pentablock copolymers. Therefore, we found that PEGE_35_-*b*-PG_30_-*b*-PEGE_35_, PEGE_20_-*b*-PG_20_-*b*-PEGE_20_-*b*-PG_20_-*b*-PEGE_20_, and PG_20_-*b*-PEGE_20_-*b*-PG_20_-*b*-PEGE_20_-*b*-PG_20_ exhibited LCST-type phase transition. For PG_x_-*b*-PEGE_y_, the phase-change behavior shows two stages. LCST-type is the first phase-change stage. UCST-like (not UCST) is the second phase-change stage, which is similar to PG_35_-*b*-PEGE_30_-*b*-PG_35_ and PEGE_15_-*b*-PG_70_-*b*-PEGE_15_. In addition, we found that PEGE homopolymers without specific α-chain ends also exhibited regular thermoresponsive properties. In general, the properties of all copolymer structures were evaluated, and the importance of copolymer disorder to thermoresponsive properties is emphasized.

## Figures and Tables

**Table 1:** Synthesis of PBnGE_x_-*stat*-PEGE_y_ and PBnGE_x_-*b*-PEGE_y_ by statistic ROP of BnGE and EGE and block ROP of BnGE as the first monomer and EGE as the second monomer, respectively, using *t*BBA as the initiator and *t*-Bu-P_4_ as the catalyst *^a^*.

Entry *^b^*	Code	[BnGE]_0_/[EGE]_0_	*M*_n,calcd_ (kg mol^−1^) *^c^*	*M*_n,NMR_ (kg mol^−1^) ^*d*^	*M*_w_/*M*_n_ *^e^*	x, y ^*f*^
1	PBnGE_x_-*stat*-PEGE_y_	10/90	11.0	10.8	1.10	10, 88
2		20/80	11.6	11.5	1.09	20, 79
3		30/70	12.2	12.2	1.09	30, 70
4		40/60	12.9	12.9	1.08	40, 60
5		50/50	13.5	13.5	1.07	50, 50
6		60/40	14.1	14.1	1.08	60, 40
7		70/30	14.7	14.7	1.08	70, 30
8		80/20	15.3	15.0	1.10	78, 20
9		90/10	16.0	15.6	1.09	88, 10
10	PBnGE_x_-*b*-PEGE_y_	10/90	11.0	10.8	1.08	10, 88
11		20/80	11.6	11.5	1.05	20, 79
12		30/70	12.2	12.2	1.04	30, 70
13		40/60	12.9	12.9	1.07	40, 60
14		50/50	13.5	13.5	1.05	50, 50

*^a^* solvent, toluene; Ar atmosphere; temperature, room temp.; [BnGE and EGE]_0_, 2.0 mol L^−1^; [*t*BBA]_0_/[*t*-Bu-P_4_]_0_, 1.0; monomer conversions were determined by ^1^H NMR in CDCl_3_. *^b^* Entries 1–9, statistic copolymerization, polymerization time, 20 h; entries 10–14, diblock copolymerizations, 1st and 2nd polymerization times*,* 20 h and 20 h; *^c^ M*_n,calcd_ = {([BnGE]_0_/[*t*BBA]_0_) × (conv. of BnGE) × (MW of BnGE)} + {([EGE]_0_/[*t*BBA]_0_) × (conv. of EGE) × (MW of EGE)]} + {(MW of *t*BBA)}. *^d^* Determined from ^1^H NMR spectra in CDCl_3_. *^e^* Determined by SEC in THF using PS standards. *^f^* Determined from the ^1^H NMR spectra in CDCl_3_.

**Table 2:** Cloud-point temperature (*T*_cp_) and average hydrodynamic radii (*D*_h_) of PG-*stat*-PEGE and PG-*b-*PEGE prepared by debenzylation of PBnGE-*stat*-PEGE and PBnGE-*b*-PEGE, respectively, using Pd/C and H_2_ *^a^*.

Entry	Starting Material	Product							
		Code	*M*_w,MALS_^*b*^ (kg mol^−1^)	*M*_w_/*M*_n_*^c^*	Yield (%)	*T*_cp_*^d^* (°C)	*D*_h_ (nm) *^e^*		
							5 °C	30 °C	90 °C
15	PBnGE_x_-*stat*-PEGE_y_	PG_10_-*stat*-PEGE_86_	9.5	1.12	93.5	30.5	10.5	-	690
16		PG_20_-*stat*-PEGE_79_	9.5	1.11	91.4	37.0	8.9	-	704
17		PG_30_-*stat*-PEGE_70_	9.4	1.13	90.1	45.4	9.6	-	725
18		PG_40_-*stat*-PEGE_60_	9.1	1.11	91.9	50.5	9.9	-	719
19		PG_50_-*stat*-PEGE_50_	8.8	1.10	93.4	56.1	10.4	-	722
20		PG_60_-*stat*-PEGE_40_	8.5	1.13	92.6	62.2	10.1	-	731
21		PG_70_-*stat*-PEGE_30_	8.2	1.11	89.3	70.4	10.8	-	754
22		PG_78_-*stat*-PEGE_20_	7.8	1.13	90.7	-	12.3	-	92
23		PG_86_-*stat*-PEGE_10_	7.4	1.12	89.0	-	15.5	-	87
24	PBnGE_x_-*b*-PEGE_y_	PG_10_-*b-*PEGE_88_	9.7	1.14	89.3	-	19.3	-	54
25		PG_20_-*b-*PEGE_79_	9.5	1.13	92.4	-	26.8	-	76
26		PG_30_-*b-*PEGE_70_	9.4	1.13	93.1	-	24.5	-	58
27		PG_40_-*b-*PEGE_60_	9.1	1.12	91.3	-	32.4	178	42
28		PG_50_-*b-*PEGE_50_	8.8	1.13	90.5	-	38.2	268	40

*^a^* Determined by the SEC equipped with a MALS in DMF in the presence of 0.01 M LiCl. *^b^* Determined by MALS in DMF containing 0.01 mol L^−1^ LiCl (dn/dc, 0.0333 mL g^−1^). *^c^* Determined by SEC in DMF using PMMA standards. *^d^* Determined by UV-vis measurements in water (10 g L^−1^). *^e^* Determined by DLS measurements in water (10 g L^−1^).

## Data Availability

The data presented in this study are available on request from the corresponding author.

## Supplementary Materials

The following are available online at https://www.mdpi.com/article/10.3390/polym13223873/s1, Table S1: Synthesis of PBnGE-*b*-PEGE-*b*-PBnGE and PEGE-*b*-PBnGE-*b*-PEGE by block ROP of BnGE and EGE using *t*BBA as the initiator and *t*-Bu-P_4_ as the catalyst, Table S2: Cloud-point temperature (*T*_cp_) and average hydrodynamic radii (*D*_h_) of PG-*b*-PEGE-*b-*PG and PEGE-*b*-PG-*b-*PEGE, prepared by the debenzylation of PBnGE-*b*-PEGE-*b-*PBnGE and PEGE-*b*-PBnGE-*b-*PEGE, respectively, Table S3: Synthesis of PBnGE-*b*-PEGE-*b*-PBnGE-*b*-PEGE-*b*-PBnGE and PEGE-*b*-PBnGE-*b*-PEGE-*b*-PBnGE-*b*-PEGE by block ROP of BnGE and EGE using *t*BBA as the initiator and *t*-Bu-P_4_ as the catalyst, Table S4: Cloud-point temperature (*T*_cp_) and average hydrodynamic radii (*D*_h_) of PG_20_-*b*-PEGE_20_-*b-*PG_20_-*b*-PEGE_20_-*b-*PG_20_ and P PEGE_20_-*b-*PG_20_-*b*-PEGE_20_-*b-*PG_20_-*b*-PEGE_20_ prepared by the debenzylation of PBnGE_20_-*b*-PEGE_20_-*b-*PBnGE_20_-*b*-PEGE_20_-*b-*PBnGE_20_ and PEGE_20_-*b-*PBnGE_20_-*b*-PEGE_20_-*b-*PBnGE_20_-*b*-PEGE_20_, respectively, Table S5: Cloud-point temperature (*T*_cp_) and average hydrodynamic radii (*D*_h_) of *t*BBA-PEGE prepared by ROP of EGE using *t*BBA as the initiator and *t*-Bu-P_4_ as the catalyst, Table S6: Cloud-point temperature (*T*_cp_) and average hydrodynamic radii (*D*_h_) of PEGE prepared by the debenzylation of *t*BBA-PEGE using Pd/C and H_2_, Figure S1: SEC trace of (a) PG_10_*-stat-*PEGE_86_, (b) PG_20_*-stat-*PEGE_79_, (c) PG_30_*-stat-*PEGE_60_, (d) PG_40_*-stat-*PEGE_60_, (e) PG_50_*-stat-*PEGE_50_ (f) PG_60_*-stat-*PEGE_40_, (g) PG_70_*-stat-*PEGE_30_, (h) PG_78_*-stat-*PEGE_20_, (i) PG_86_*-stat-*PEGE_10_ in DMF, Figure S2: ^1^H NMR spectra of PEGE_15_-*b*-PG_70_-*b*-PEGE_15_ at 23 °C, 33 °C, 43 °C, and 23 °C (cooling to 23 °C) in D_2_O, Figure S3: Hydrodynamic diameter distribution of PG_15_-*b*-PEGE_70_-*b-*PG_15_ at (a) 20 °C, and (b) 60 °C (scattering angle, 90°), Figure S4: (a) ^1^H NMR and (b) ^13^C NMR spectra of *t*BBA-PEGE_25_ in MeOD (the symbol * refers to solvent peaks), Figure S5: MALDI-TOF MS spectrum of *t*BBA-PBnGE_25_, Figure S6: ^1^H NMR and spectra of PEGE_50_ in MeOD (the symbol * refers to solvent peaks), Figure S7: SEC trace of PEGE_25,50,75, 100_ in DMF, Figure S8: Hydrodynamic diameter distribution of PEGE_50_ at (a) 10 °C and (b) 45 °C (scattering angle, 90°).
