# Supplementary material for: Precise Synthesis and Thermoresponsive Property of Poly(ethyl glycidyl ether) and Its Block and Statistic Copolymers with Poly(glycidol)
Source: Polymers (Basel). 2021 Nov 9;13(22):3873. doi: 10.3390/polym13223873 (PMC8623496; doi:10.3390/polym13223873)
Supplement: Supplementary file 1 [file polymers-13-03873-s001.zip › polymers-1445596-supplementary.pdf]

polymers MDPI

Supplementary Material: Precise Synthesis and Thermoresponsive Property of Poly(ethyl glycidyl ether) and Its Block and Statistic Copolymers with Poly(glycidol)

Tingyu He, Yanqiu Wang, Atsushi Narumi, Liang Xu, Shin-ichiro Sato, Xiande Shen, and Toyoji Kakuchi

1. Synthesis of PEGE, PG-*b*-PEGE-*b*-PG, and PG-*b*-PEGE-*b*-PG-*b*-PEGE-*b*-PG

Synthesis of *t*BBA-PEGE. A typical procedure for the polymerization of EGE is described as follows: To a solution of *t*BBA (19.7 mg, 120 μmol) in 2.2 mL dry toluene, 0.15 mL of *t*-Bu-P4 (120 μmol as 0.8 M solution in n-hexane) and EGE (0.613 g, 6 mmol) were added in this order. The color of the solution changed from light yellow to dark yellow, indicating that the polymerization was successfully initiated. After completion of EGE polymerization (20 h, determined the conversion of EGE by ^{1}H NMR measurements), the polymerization was then quenched by adding a small amount of benzoic acid mixture to the polymerization solution. Aliquots were removed from the polymerization mixture to determine the conversion of EGE by ^{1}H NMR measurements. The obtained polymer was purified through neutralized aluminum oxide to give tBBA-PEGE50 as a colorless and viscous liquid. Yield, 93.7%; $M_{n,NMR}$, 5.2 kg mol^{-1}; M_w/M_n, 1.06.

Synthesis of PEGE. A typical deprotection procedure is described as follows. Palladium on carbon (10% Pd/C; 100 mg) was added to a solution of *t*BBA-PEGE50 (100 mg; 19.2 μmol) in a mixture of methanol and CH2Cl2 (1/1, v/v; 10.00 mL), and the whole mixture was stirred under a hydrogen atmosphere (3-5MPa) for 24 h. After removing the Pd/C catalyst by filtration using celite, the solution was evaporated to obtain PEGE50 as a colorless and viscous liquid. Yield, 93.2%; $M_{w,MALS}$, 5.0 kg mol−1; M_w/M_n, 1.08.

Synthesis of PBnGE-*b*-PEGE-*b*-PBnGE. A typical procedure for the triblock copolymerization of BnGE and EGE is described as follows: To a solution of *t*BBA (13.14 mg, 80 μmol) in 2.96 mL dry toluene, 0.10 mL of *t*-Bu-P$_4$ (80 μmol as 0.8 M solution in *n*-hexane) and BnGE (0.46 g, 2.8 mmol) were added in this order. The color of the solution changed from light yellow to dark yellow, indicating that the polymerization was successfully initiated. After completion of BnGE polymerization (20 h, determined the conversion of BnGE by ^{1}H NMR measurements), EGE (0.25 g, 2.4 mmol) was further added to the polymerization mixture to continue the block copolymerization for 20 h. After completion of EGE polymerization, BnGE (0.46 g, 2.8 mmol) was further added to the polymerization mixture to continue the block copolymerization for 20 h. The polymerization was then quenched by adding a small amount of benzoic acid mixture to the polymerization solution. Aliquots were removed from the polymerization mixture to determine the conversion of BnGE and EGE by ^{1}H NMR measurements. The obtained polymer was purified through neutralized aluminum oxide to give PBnGE$_{35}$-*b*-PEGE$_{30}$-*b*-PBnGE$_{35}$ as a colorless and viscous liquid. Yield, 90.7%; $M_{n,NMR}$, 14.4 kg mol^{-1}; M_w/M_n, 1.13.

Synthesis of PG-*b*-PEGE-*b*-PG. A typical deprotection procedure is described as follows. Palladium on carbon (10% Pd/C; 100 mg) was added to a solution of PBnGE$_{35}$-*b*-PEGE$_{30}$-*b*-PBnGE$_{35}$ (100 mg; 6.94 μmol) in a mixture of methanol and CH_2Cl_2 (1/1, v/v; 10.00 mL), and the whole mixture was stirred under a hydrogen atmosphere (3-5MPa) for 24 h. After removing the Pd/C catalyst by filtration using celite, the solution was evaporated to obtain PG$_{35}$-*b*-PEGE$_{30}$-*b*-PG$_{35}$ as a colorless and viscous liquid. Yield, 89.6%; $M_{w,MALS}$, 8.1 kg mol^{-1}; M_w/M_n, 1.15.

Synthesis of PBnGE-*b*-PEGE-*b*-PBnGE-*b*-PEGE-*b*-PBnGE. A typical procedure for the pentablock copolymerization of BnGE and EGE is described as follows: A typical procedure for the triblock copolymerization of BnGE and EGE is described as follows: To

a solution of *t*BBA (16.4 mg, 100 μmol) in 2.46 mL dry toluene, 0.125 mL of *t*-Bu-P_4 (120 μmol as 0.8 M solution in *n*-hexane) and BnGE (0.328 g, 2 mmol) were added in this order. The color of the solution changed from light yellow to dark yellow, indicating that the polymerization was successfully initiated. After completion of BnGE polymerization (20 h, determined the conversion of BnGE by ^{1}H NMR measurements), EGE (0.204 g, 2 mmol) was further added to the polymerization mixture to continue the block copolymerization for 20 h. After completion of EGE polymerization, BnGE (0.328 g, 2 mmol) was further added to the polymerization mixture to continue the block copolymerization for 20 h. When the conversion of BnGE determined by ^{1}H NMR measurements reached 99.9%, add EGE (0.204 g, 2 mmol) again. After 20 h, continue to add the same amount of BnGE as the last time. The polymerization was then quenched by adding a small amount of benzoic acid mixture to the polymerization solution. Aliquots were removed from the polymerization mixture to determine the conversion of BnGE and EGE by ^{1}H NMR measurements. The obtained polymer was purified through neutralized aluminum oxide to give $PBnGE_{20}$-*b*-$PEGE_{20}$-*b*-$PBnGE_{20}$-*b*-$PEGE_{20}$-*b*-$PBnGE_{20}$ as a colorless and viscous liquid. Yield, 89.7%; $M_{n,NMR}$, 13.8 kg mol^{-1}; M_w/M_n, 1.12.

Synthesis of PG-*b*-PEGE-*b*-PG-*b*-PEGE-*b*-PG. A typical deprotection procedure is described as follows. Palladium on carbon (10% Pd/C; 100 mg) was added to a solution of $PBnGE_{20}$-*b*-$PEGE_{20}$-*b*-$PBnGE_{20}$-*b*-$PEGE_{20}$-*b*-$PBnGE_{20}$ (100 mg; 7.25 μmol) in a mixture of methanol and CH_2Cl_2 (1/1, v/v; 10.00 mL), and the whole mixture was stirred under a hydrogen atmosphere (3-5MPa) for 24 h. After removing the Pd/C catalyst by filtration using celite, the solution was evaporated to obtain PG_{20}-*b*-$PEGE_{20}$-*b*-PG_{20}-*b*-$PEGE_{20}$-*b*-PG_{20} as a colorless and viscous liquid. Yield, 88.1%; $M_{w,MALS}$, 8.4 kg mol^{-1}; M_w/M_n, 1.14.

2. Tables S1 – S6

Table S1. Synthesis of PBnGE-*b*-PEGE-*b*-PBnGE and PEGE-*b*-PBnGE-*b*-PEGE by block ROP of BnGE and EGE using tBBA as the initiator and t-Bu-P4 as the catalyst [a].

sample	$[M_{1st}]/[M_{2nd}]/[M_{3rd}]$	$M_{n,calcd}$ [b] /kg mol^{-1}	$M_{n,NMR}$ [c] / kg mol^{-1}	DP_{1st}, DP_{2nd}, DP_{3rd} [d]	M_w/M_n [e]
$PBnGE_{15}$-*b*-$PEGE_{70}$-*b*-$PBnGE_{15}$	15/70/15	12.1	12.0	15, 69.5, 15	1.12
$PBnGE_{35}$-*b*-$PEGE_{30}$-*b*-$PBnGE_{35}$	35/30/35	14.6	13.2	34.7, 30, 34.8	1.13
$PEGE_{15}$-*b*-$PBnGE_{70}$-*b*-$PEGE_{15}$	15/70/15	14.6	14.5	15, 69.6, 15	1.12
$PEGE_{35}$-*b*-$PBnGE_{30}$-*b*-$PEGE_{35}$	35/30/35	12.0	12.0	34.6, 30, 34.7	1.14

[a] Solvent, toluene; Ar atmosphere; temperature, room temp.; polymerization time, 20 h (1st ROP) + 20 h (2nd ROP) + 20 h (3rd ROP); $[M]_0$ = 2.0 mol L^{-1}; $[tBBA]_0/[t\text{-Bu-}P_4]_0$ = 1.0; monomer conversions were determined by ^{1}H NMR in $CDCl_3$. [b] $M_{n,calcd}$ = {($[M_{1st}]_0/[tBBA]_0$) × (conv. for the 1st ROP) × (MW of M_{1st})} + {($[M_{2nd}]_0/[tBBA]_0$) × (conv. for the 2nd ROP) × (MW of M_{2nd})]} + {($[M_{3rd}]_0/[tBBA]_0$) × (conv. for the 3rd ROP) × (MW of M_{3rd})]} + {(MW of *t*BBA)}. [c] The number average molar mass determined from ^{1}H NMR spectra in $CDCl_3$. [d] Determined from the ^{1}H NMR spectra in $CDCl_3$. [e] Determined by SEC in THF using PS standards.

Table S2. Cloud point temperature (T_{cp}) and average hydrodynamic radii (D_h) of PG-*b*-PEGE-*b*-PG and PEGE-*b*-PG-*b*-PEGE prepared by the debenzylation of PBnGE-*b*-PEGE-*b*-PBnGE and PEGE-*b*-PBnGE-*b*-PEGE, respectively [a].

Starting material	Product				D_h /nm [e]		
	Code	$M_{w,MALS}$ [b] kg mol^{-1}	M_w/M_n [c]	T_{cp} °C [d]	5 °C	20 °C	60 °C
$PBnGE_{15}$-*b*-$PEGE_{70}$-*b*-$PBnGE_{15}$	PG_{15}-*b*-$PEGE_{70}$-*b*-PG_{15}	9.2	1.14	-	28	-	240
$PBnGE_{35}$-*b*-$PEGE_{30}$-*b*-$PBnGE_{35}$	PG_{35}-*b*-$PEGE_{30}$-*b*-PG_{35}	8.1	1.15	-	39	443	98
$PEGE_{15}$-*b*-$PBnGE_{70}$-*b*-$PEGE_{15}$	$PEGE_{15}$-*b*-PG_{70}-*b*-$PEGE_{15}$	8.1	1.14	-	33	396	117
$PEGE_{35}$-*b*-$PBnGE_{30}$-*b*-$PEGE_{35}$	$PEGE_{35}$-*b*-PG_{30}-*b*-$PEGE_{35}$	9.3	1.14	50.6	23	-	545

[a] Determined by the SEC equipped with a MALS in DMF in the presence of 0.01 M LiCl. [b] Determined by MALS in DMF containing 0.01 mol L^{-1} LiCl. [c] Determined by SEC in DMF using PMMA standards. [d] Determined by UV-vis measurements in water (10 g L^{-1}). [e] Determined by DLS measurements in water (10 g L^{-1}).

Table S3. Synthesis of PBnGE-*b*-PEGE-*b*-PBnGE-*b*-PEGE-*b*-PBnGE and PEGE-*b*-PBnGE-*b*-PEGE-*b*-PBnGE-*b*-PEGE by block ROP of BnGE and EGE using tBBA as the initiator and t-Bu-P4 as the catalyst [a].

sample	$M_{n,calcd}$ [b] (kg mol^{-1})	$M_{n,NMR}$ [c] (kg mol^{-1})	M_w/M_n [d]
PBnGE$_{20}$-*b*-PEGE$_{20}$-*b*-PBnGE$_{20}$-*b*-PEGE$_{20}$-*b*-PBnGE$_{20}$	14.1	13.8	1.12
PEGE$_{20}$-*b*-PBnGE$_{20}$ *b*-PEGE$_{20}$-*b*-PBnGE$_{20}$ *b*-PEGE$_{20}$	12.7	12.5	1.12

[a] Solvent, toluene; room temperature; Ar atmosphere; polymerization time, 20 h (1st ROP) + 20 h (2nd ROP) + 20 h (3rd ROP) + 20 h (4th ROP) + 20 h (5th ROP); [BnGE + EGE]$_0$ = 2.0 mol L^{-1}; [*t*BBA]$_0$/[*t*-Bu-P$_4$]$_0$ = 1.0; monomer conversions were >99% determined by ^{1}H NMR in CDCl$_3$. [b] $M_{n,calcd}$ = {([M$_{1st}$]$_0$/[*t*BBA]$_0$) × (conv. for the 1st ROP) × (MW of M$_{1st}$)} + {([M$_{2nd}$]$_0$/[*t*BBA]$_0$) × (conv. for the 2nd ROP) × (MW of M$_{2nd}$)]} + {([M$_{3rd}$]$_0$/[*t*BBA]$_0$) × (conv. for the 3rd ROP) × (MW of M$_{3rd}$)]} + {([M$_{4th}$]$_0$/[*t*BBA]$_0$) × (conv. for the 4th ROP) × (MW of M$_{4th}$)]} + {([M$_{5th}$]$_0$/[*t*BBA]$_0$) × (conv. for the 5th ROP) × (MW of M$_{5th}$)]} + {(MW of *t*BBA)}. [c] Determined from ^{1}H NMR spectra in CDCl$_3$. [d] Determined by SEC in THF using PS standards.

Table S4. Cloud point temperature (T_{cp}) and average hydrodynamic radii (D_h) of PG$_{20}$-*b*-PEGE$_{20}$-*b*-PG$_{20}$-*b*-PEGE$_{20}$-*b*-PG$_{20}$ and P PEGE$_{20}$-*b*-PG$_{20}$-*b*-PEGE$_{20}$-*b*-PG$_{20}$-*b*-PEGE$_{20}$ prepared by the debenzylation of PBnGE$_{20}$-*b*-PEGE$_{20}$-*b*-PBnGE$_{20}$-*b*-PEGE$_{20}$-*b*-PBnGE$_{20}$ and PEGE$_{20}$-*b*-PBnGE$_{20}$-*b*-PEGE$_{20}$-*b*-PBnGE$_{20}$-*b*-PEGE$_{20}$, respectively [a].

Starting material	Product					
	Code	$M_{w,mALS}$ [b] (kg mol^{-1})	M_w/M_n [c]	T_{cp} (°C) [d]	D_h(nm) [e] 25 °C 35°C	T_{cp}
PBnGE$_{20}$-*b*-PEGE$_{20}$-*b*-PBnGE$_{20}$-*b*-PEGE$_{20}$-*b*-PBnGE$_{20}$	PG$_{20}$-*b*-PEGE$_{20}$-*b*-PG$_{20}$-*b*-PEGE$_{20}$-*b*-PG$_{20}$	8.4	1.14	61.7	18	688
PEGE$_{20}$-*b*-PBnGE$_{20}$-*b*-PEGE$_{20}$-*b*-PBnGE$_{20}$-*b*-PEGE$_{20}$	PEGE$_{20}$-*b*-PG$_{20}$-*b*-PEGE$_{20}$-*b*-PG$_{20}$-*b*-PEGE$_{20}$	18.5	1.14	48.3	15	663

[a] Determined by the SEC equipped with a MALS in DMF in the presence of 0.01 M LiCl. [b] Determined by MALS in DMF containing 0.01 mol L^{-1} LiCl. [c] Determined by SEC in DMF using PMMA standards. [d] Determined by UV-vis measurements in water (10 g L^{-1}). [e] Determined by DLS measurements in water (10 g L^{-1}).

Table S5. Cloud point temperature (T_{cp}) and average hydrodynamic radii (D_h) of *t*BBA-PEGE prepared by ROP of EGE using *t*BBA as the initiator and *t*-Bu-P$_4$ as the catalyst [a].

sample	Conv. [b] / %	$M_{n,calcd}$ [c] / kg mol^{-1}	$M_{n,NMR}$ [d] / kg mol^{-1} (DP)	M_w/M_n [e]	T_{cp} (°C) [f]	D_h (nm) [g] 5 °C	T_{cp}
*t*BBA-PEGE$_{25}$	>99	2.7	2.7 (24.9)	1.05	12.1	12	390
*t*BBA-PEGE$_{50}$	>99	5.3	5.2 (49.7)	1.06	11.5	18	423
*t*BBA-PEGE$_{75}$	>99	7.8	7.7 (74.2)	1.06	10.4	21	533
*t*BBA-PEGE$_{100}$	98.9	10.4	10.2 (98.6)	1.07	9.1	24	595

[a] Solvent, toluene; room temperature; Ar atmosphere; polymerization time, 20 h; EGE]$_0$ = 2.0 mol L^{-1}; [*t*BBA]$_0$/[*t*-Bu-P$_4$]$_0$ = 1.0; Determined by ^{1}H NMR in CDCl$_3$. [c] $M_{n,calcd}$ = {([EGE]$_0$/[*t*BBA]$_0$) × (conv.) × (MW of EGE)} + {(MW of *t*BBA)}. [d] Determined from ^{1}H NMR spectra in CDCl$_3$. [e] Determined by SEC in THF using PS standards. [f] Determined by UV-vis measurements in water (10 g L^{-1}). [g] Determined by DLS measurements in water (10 g L^{-1}).

Table S6. Cloud point temperature (T_{cp}) and average hydrodynamic radii (D_h) of PEGE prepared by the debenzylation of *t*BBA-PEGE using Pd/C and H$_2$ [a].

Starting material	Product					
	Code	$M_{w,MALS}$ [b] (kg mol^{-1})	M_w/M_n [c]	T_{cp} [d] (°C)	D_h [e] (nm) 10 °C	T_{cp}
*t*BBA-PEGE$_{25}$	PEGE$_{25}$	2.7	1.08	32.5	14	402
*t*BBA-PEGE$_{50}$	PEGE$_{50}$	5.1	1.08	29.8	16	449
*t*BBA-PEGE$_{75}$	PEGE$_{75}$	7.6	1.09	27.1	20	558
*t*BBA-PEGE$_{100}$	PEGE$_{100}$	10.1	1.09	25.2	22	610

[a] Solvent, toluene; Ar atmosphere; room temperature; polymerization time, 20 h; [EGE]$_0$ = 2.0 mol L^{-1}; [*t*BBA]$_0$/[*t*-Bu-P$_4$]$_0$ = 1.0; monomer conversions were >99% determined by ^{1}H NMR in CDCl$_3$. [c] Determined by SEC in THF using PS standards. [d] Determined by UV-vis measurements in water (10 g L^{-1}). [e] Determined by DLS measurements in water (10 g L^{-1}).

3. Figures S1 – S7

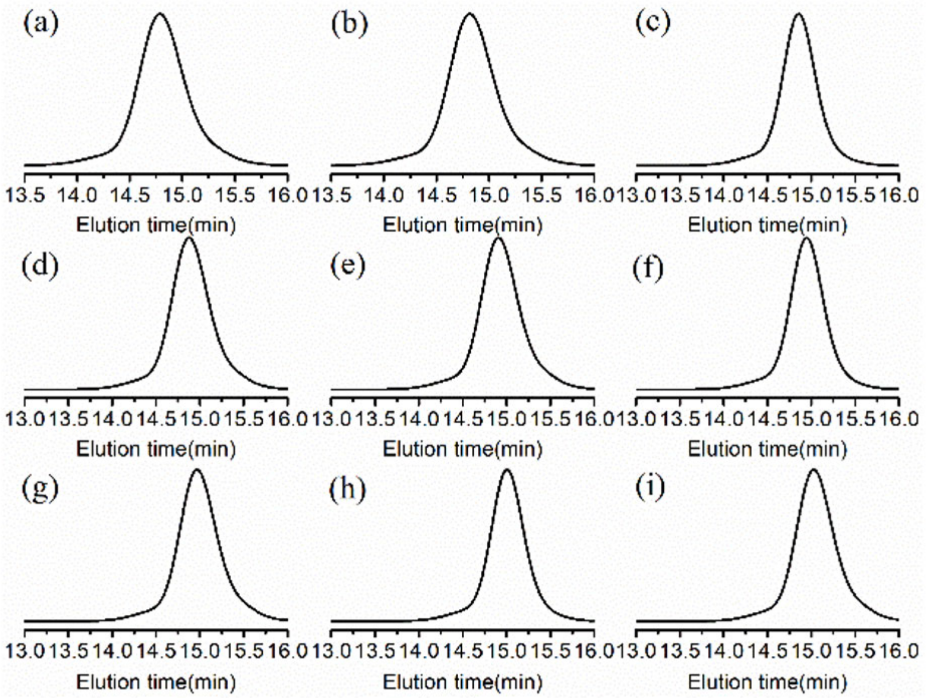

Figure S1. SEC trace of (**a**) PG_{10}-*stat*-$PEGE_{86}$, (**b**) PG_{20}-*stat*-$PEGE_{79}$, (**c**) PG_{30}-*stat*-$PEGE_{60}$, (**d**) PG_{40}-*stat*-$PEGE_{60}$, (**e**) PG_{50}-*stat*-$PEGE_{50}$ (**f**) PG_{60}-*stat*-$PEGE_{40}$, (**g**) PG_{70}-*stat*-$PEGE_{30}$, (**h**) PG_{78}-*stat*-$PEGE_{20}$, (**i**) PG_{86}-*stat*-$PEGE_{10}$ in DMF.

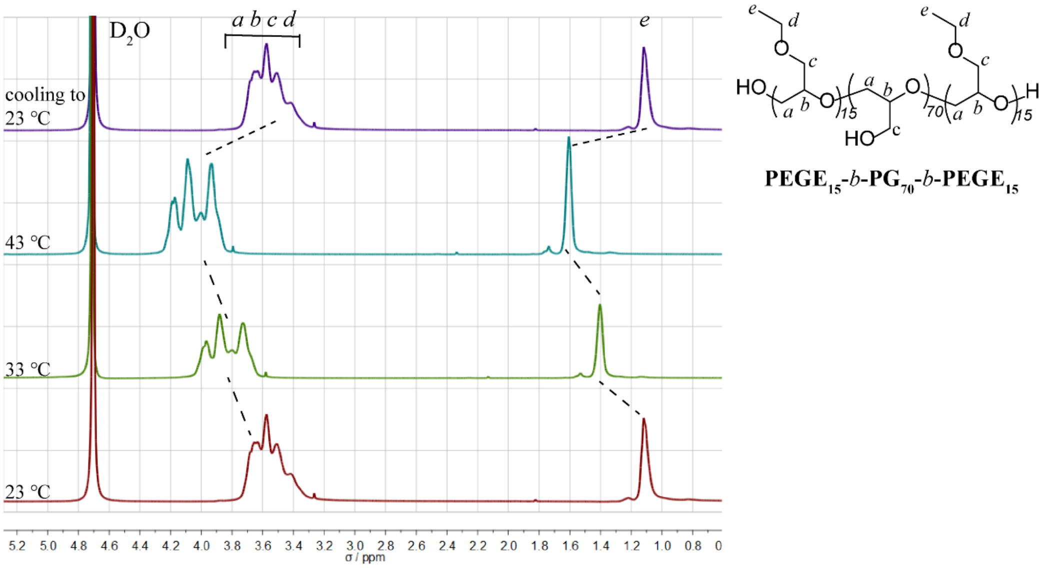

Figure S2. ¹H NMR spectra of $PEGE_{15}$-*b*-PG_{70}-*b*-$PEGE_{15}$ at 23 °C, 33 °C, 43 °C, and 23 °C (cooling to 23 °C) in D_2O.

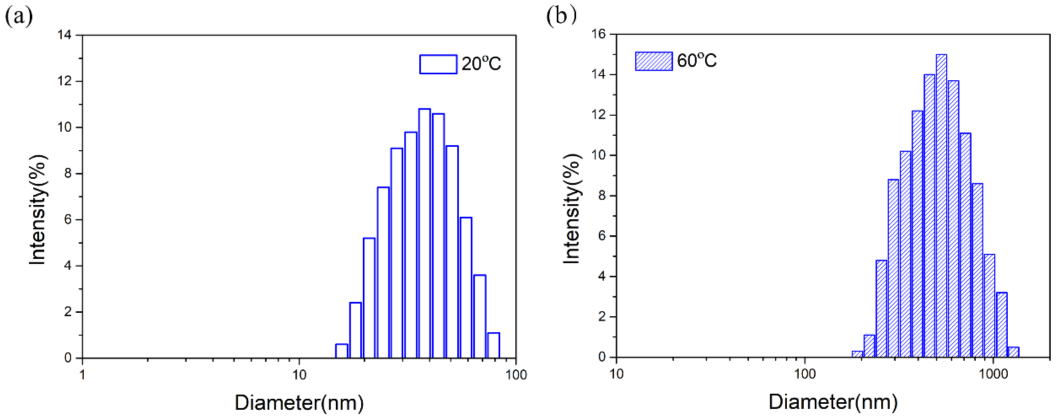

Figure S3. Hydrodynamic diameter distribution of PG_{15}-*b*-$PEGE_{70}$-*b*-PG_{15} at (**a**) 20 °C, and (**b**) 60 °C (scattering angle, 90 °).

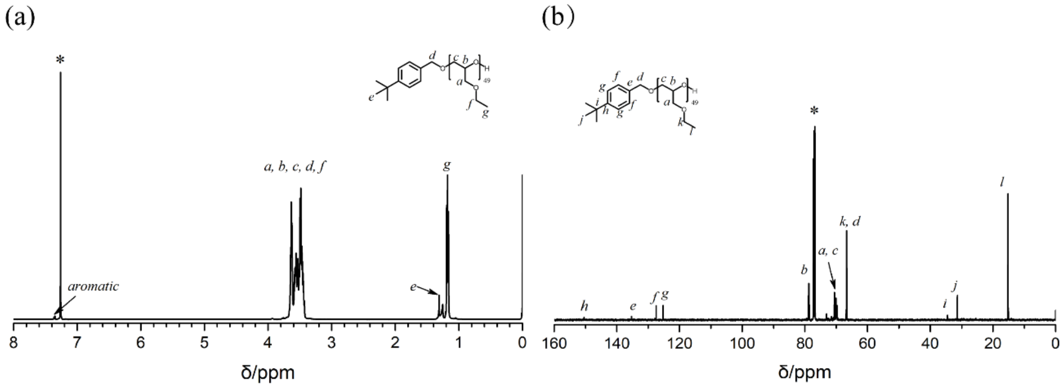

Figure S4. (**a**)^{1}H NMR and (**b**) ^{13}C NMR spectra of *t*BBA-$PEGE_{25}$ in MeOD (the symbol * refers to solvent peaks).

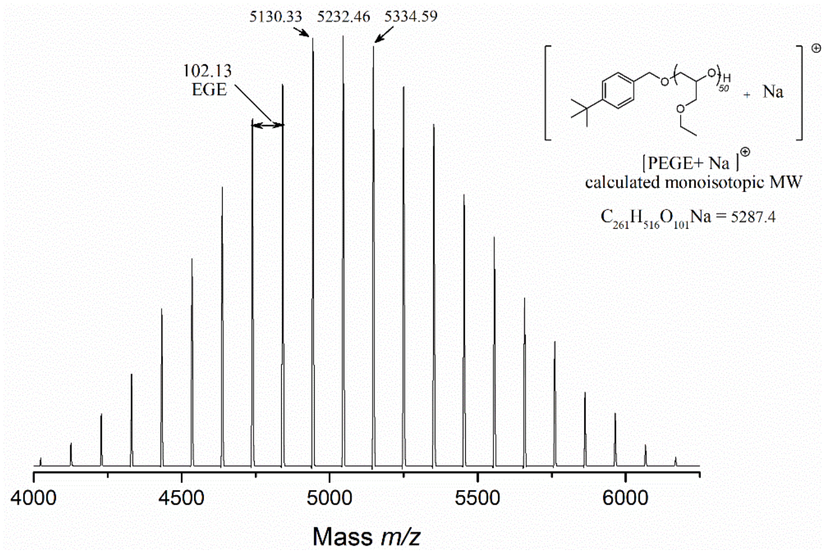

Figure S5. MALDI-TOF MS spectrum of *t*BBA-$PBnGE_{25}$.

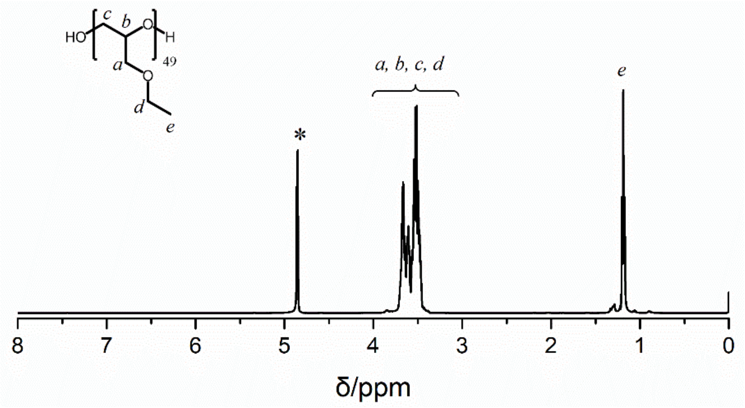

Figure S6. ^{1}H NMR and spectra of $PEGE_{50}$ in MeOD (the symbol * refers to solvent peaks).

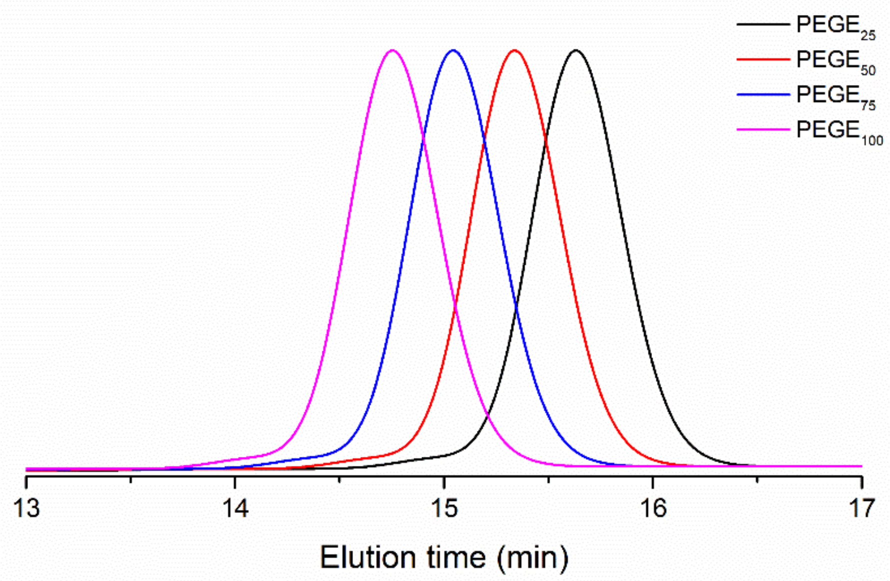

Figure S7. SEC trace of $PEGE_{25,50,75,\ 100}$ in DMF.

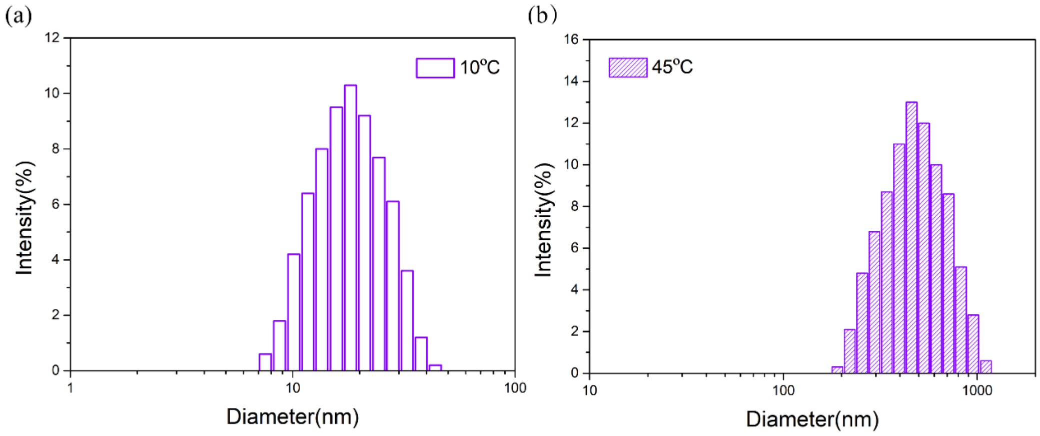

Figure S8. Hydrodynamic diameter distribution of $PEGE_{50}$ at (**a**) 10 °C and (**b**) 45 °C (scattering angle, 90°).
